# Search for heavy long-lived multi-charged particles in *pp* collisions at $\sqrt{s}=8$ TeV using the ATLAS detector

**DOI:** 10.1140/epjc/s10052-015-3534-2

**Published:** 2015-08-08

**Authors:** G. Aad, B. Abbott, J. Abdallah, O. Abdinov, R. Aben, M. Abolins, O. S. AbouZeid, H. Abramowicz, H. Abreu, R. Abreu, Y. Abulaiti, B. S. Acharya, L. Adamczyk, D. L. Adams, J. Adelman, S. Adomeit, T. Adye, A. A. Affolder, T. Agatonovic-Jovin, J. A. Aguilar-Saavedra, S. P. Ahlen, F. Ahmadov, G. Aielli, H. Akerstedt, T. P. A. Åkesson, G. Akimoto, A. V. Akimov, G. L. Alberghi, J. Albert, S. Albrand, M. J. Alconada Verzini, M. Aleksa, I. N. Aleksandrov, C. Alexa, G. Alexander, T. Alexopoulos, M. Alhroob, G. Alimonti, L. Alio, J. Alison, S. P. Alkire, B. M. M. Allbrooke, P. P. Allport, A. Aloisio, A. Alonso, F. Alonso, C. Alpigiani, A. Altheimer, B. Alvarez Gonzalez, D. Álvarez Piqueras, M. G. Alviggi, K. Amako, Y. Amaral Coutinho, C. Amelung, D. Amidei, S. P. Amor Dos Santos, A. Amorim, S. Amoroso, N. Amram, G. Amundsen, C. Anastopoulos, L. S. Ancu, N. Andari, T. Andeen, C. F. Anders, G. Anders, J. K. Anders, K. J. Anderson, A. Andreazza, V. Andrei, S. Angelidakis, I. Angelozzi, P. Anger, A. Angerami, F. Anghinolfi, A. V. Anisenkov, N. Anjos, A. Annovi, M. Antonelli, A. Antonov, J. Antos, F. Anulli, M. Aoki, L. Aperio Bella, G. Arabidze, Y. Arai, J. P. Araque, A. T. H. Arce, F. A. Arduh, J-F. Arguin, S. Argyropoulos, M. Arik, A. J. Armbruster, O. Arnaez, V. Arnal, H. Arnold, M. Arratia, O. Arslan, A. Artamonov, G. Artoni, S. Asai, N. Asbah, A. Ashkenazi, B. Åsman, L. Asquith, K. Assamagan, R. Astalos, M. Atkinson, N. B. Atlay, B. Auerbach, K. Augsten, M. Aurousseau, G. Avolio, B. Axen, M. K. Ayoub, G. Azuelos, M. A. Baak, A. E. Baas, C. Bacci, H. Bachacou, K. Bachas, M. Backes, M. Backhaus, P. Bagiacchi, P. Bagnaia, Y. Bai, T. Bain, J. T. Baines, O. K. Baker, P. Balek, T. Balestri, F. Balli, E. Banas, Sw. Banerjee, A. A. E. Bannoura, H. S. Bansil, L. Barak, E. L. Barberio, D. Barberis, M. Barbero, T. Barillari, M. Barisonzi, T. Barklow, N. Barlow, S. L. Barnes, B. M. Barnett, R. M. Barnett, Z. Barnovska, A. Baroncelli, G. Barone, A. J. Barr, F. Barreiro, J. Barreiro Guimarães da Costa, R. Bartoldus, A. E. Barton, P. Bartos, A. Basalaev, A. Bassalat, A. Basye, R. L. Bates, S. J. Batista, J. R. Batley, M. Battaglia, M. Bauce, F. Bauer, H. S. Bawa, J. B. Beacham, M. D. Beattie, T. Beau, P. H. Beauchemin, R. Beccherle, P. Bechtle, H. P. Beck, K. Becker, M. Becker, S. Becker, M. Beckingham, C. Becot, A. J. Beddall, A. Beddall, V. A. Bednyakov, C. P. Bee, L. J. Beemster, T. A. Beermann, M. Begel, J. K. Behr, C. Belanger-Champagne, W. H. Bell, G. Bella, L. Bellagamba, A. Bellerive, M. Bellomo, K. Belotskiy, O. Beltramello, O. Benary, D. Benchekroun, M. Bender, K. Bendtz, N. Benekos, Y. Benhammou, E. Benhar Noccioli, J. A. Benitez Garcia, D. P. Benjamin, J. R. Bensinger, S. Bentvelsen, L. Beresford, M. Beretta, D. Berge, E. Bergeaas Kuutmann, N. Berger, F. Berghaus, J. Beringer, C. Bernard, N. R. Bernard, C. Bernius, F. U. Bernlochner, T. Berry, P. Berta, C. Bertella, G. Bertoli, F. Bertolucci, C. Bertsche, D. Bertsche, M. I. Besana, G. J. Besjes, O. Bessidskaia Bylund, M. Bessner, N. Besson, C. Betancourt, S. Bethke, A. J. Bevan, W. Bhimji, R. M. Bianchi, L. Bianchini, M. Bianco, O. Biebel, S. P. Bieniek, M. Biglietti, J. Bilbao De Mendizabal, H. Bilokon, M. Bindi, S. Binet, A. Bingul, C. Bini, C. W. Black, J. E. Black, K. M. Black, D. Blackburn, R. E. Blair, J.-B. Blanchard, J. E. Blanco, T. Blazek, I. Bloch, C. Blocker, W. Blum, U. Blumenschein, G. J. Bobbink, V. S. Bobrovnikov, S. S. Bocchetta, A. Bocci, C. Bock, M. Boehler, J. A. Bogaerts, A. G. Bogdanchikov, C. Bohm, V. Boisvert, T. Bold, V. Boldea, A. S. Boldyrev, M. Bomben, M. Bona, M. Boonekamp, A. Borisov, G. Borissov, S. Borroni, J. Bortfeldt, V. Bortolotto, K. Bos, D. Boscherini, M. Bosman, J. Boudreau, J. Bouffard, E. V. Bouhova-Thacker, D. Boumediene, C. Bourdarios, N. Bousson, A. Boveia, J. Boyd, I. R. Boyko, I. Bozic, J. Bracinik, A. Brandt, G. Brandt, O. Brandt, U. Bratzler, B. Brau, J. E. Brau, H. M. Braun, S. F. Brazzale, K. Brendlinger, A. J. Brennan, L. Brenner, R. Brenner, S. Bressler, K. Bristow, T. M. Bristow, D. Britton, D. Britzger, F. M. Brochu, I. Brock, R. Brock, J. Bronner, G. Brooijmans, T. Brooks, W. K. Brooks, J. Brosamer, E. Brost, J. Brown, P. A. Bruckman de Renstrom, D. Bruncko, R. Bruneliere, A. Bruni, G. Bruni, M. Bruschi, L. Bryngemark, T. Buanes, Q. Buat, P. Buchholz, A. G. Buckley, S. I. Buda, I. A. Budagov, F. Buehrer, L. Bugge, M. K. Bugge, O. Bulekov, D. Bullock, H. Burckhart, S. Burdin, B. Burghgrave, S. Burke, I. Burmeister, E. Busato, D. Büscher, V. Büscher, P. Bussey, J. M. Butler, A. I. Butt, C. M. Buttar, J. M. Butterworth, P. Butti, W. Buttinger, A. Buzatu, A. R. Buzykaev, S. Cabrera Urbán, D. Caforio, V. M. Cairo, O. Cakir, P. Calafiura, A. Calandri, G. Calderini, P. Calfayan, L. P. Caloba, D. Calvet, S. Calvet, R. Camacho Toro, S. Camarda, P. Camarri, D. Cameron, L. M. Caminada, R. Caminal Armadans, S. Campana, M. Campanelli, A. Campoverde, V. Canale, A. Canepa, M. Cano Bret, J. Cantero, R. Cantrill, T. Cao, M. D. M. Capeans Garrido, I. Caprini, M. Caprini, M. Capua, R. Caputo, R. Cardarelli, T. Carli, G. Carlino, L. Carminati, S. Caron, E. Carquin, G. D. Carrillo-Montoya, J. R. Carter, J. Carvalho, D. Casadei, M. P. Casado, M. Casolino, E. Castaneda-Miranda, A. Castelli, V. Castillo Gimenez, N. F. Castro, P. Catastini, A. Catinaccio, J. R. Catmore, A. Cattai, J. Caudron, V. Cavaliere, D. Cavalli, M. Cavalli-Sforza, V. Cavasinni, F. Ceradini, B. C. Cerio, K. Cerny, A. S. Cerqueira, A. Cerri, L. Cerrito, F. Cerutti, M. Cerv, A. Cervelli, S. A. Cetin, A. Chafaq, D. Chakraborty, I. Chalupkova, P. Chang, B. Chapleau, J. D. Chapman, D. G. Charlton, C. C. Chau, C. A. Chavez Barajas, S. Cheatham, A. Chegwidden, S. Chekanov, S. V. Chekulaev, G. A. Chelkov, M. A. Chelstowska, C. Chen, H. Chen, K. Chen, L. Chen, S. Chen, X. Chen, Y. Chen, H. C. Cheng, Y. Cheng, A. Cheplakov, E. Cheremushkina, R. Cherkaoui El Moursli, V. Chernyatin, E. Cheu, L. Chevalier, V. Chiarella, J. T. Childers, G. Chiodini, A. S. Chisholm, R. T. Chislett, A. Chitan, M. V. Chizhov, K. Choi, S. Chouridou, B. K. B. Chow, V. Christodoulou, D. Chromek-Burckhart, M. L. Chu, J. Chudoba, A. J. Chuinard, J. J. Chwastowski, L. Chytka, G. Ciapetti, A. K. Ciftci, D. Cinca, V. Cindro, I. A. Cioara, A. Ciocio, Z. H. Citron, M. Ciubancan, A. Clark, B. L. Clark, B. L. Clark, P. J. Clark, R. N. Clarke, W. Cleland, C. Clement, Y. Coadou, M. Cobal, A. Coccaro, J. Cochran, L. Coffey, J. G. Cogan, B. Cole, S. Cole, A. P. Colijn, J. Collot, T. Colombo, G. Compostella, P. Conde Muiño, E. Coniavitis, S. H. Connell, I. A. Connelly, S. M. Consonni, V. Consorti, S. Constantinescu, C. Conta, G. Conti, F. Conventi, M. Cooke, B. D. Cooper, A. M. Cooper-Sarkar, T. Cornelissen, M. Corradi, F. Corriveau, A. Corso-Radu, A. Cortes-Gonzalez, G. Cortiana, G. Costa, M. J. Costa, D. Costanzo, D. Côté, G. Cottin, G. Cowan, B. E. Cox, K. Cranmer, G. Cree, S. Crépé-Renaudin, F. Crescioli, W. A. Cribbs, M. Crispin Ortuzar, M. Cristinziani, V. Croft, G. Crosetti, T. Cuhadar Donszelmann, J. Cummings, M. Curatolo, C. Cuthbert, H. Czirr, P. Czodrowski, S. D’Auria, M. D’Onofrio, M. J. Da Cunha Sargedas De Sousa, C. Da Via, W. Dabrowski, A. Dafinca, T. Dai, O. Dale, F. Dallaire, C. Dallapiccola, M. Dam, J. R. Dandoy, N. P. Dang, A. C. Daniells, M. Danninger, M. Dano Hoffmann, V. Dao, G. Darbo, S. Darmora, J. Dassoulas, A. Dattagupta, W. Davey, C. David, T. Davidek, E. Davies, M. Davies, P. Davison, Y. Davygora, E. Dawe, I. Dawson, R. K. Daya-Ishmukhametova, K. De, R. de Asmundis, S. De Castro, S. De Cecco, N. De Groot, P. de Jong, H. De la Torre, F. De Lorenzi, L. De Nooij, D. De Pedis, A. De Salvo, U. De Sanctis, A. De Santo, J. B. De Vivie De Regie, W. J. Dearnaley, R. Debbe, C. Debenedetti, D. V. Dedovich, I. Deigaard, J. Del Peso, T. Del Prete, D. Delgove, F. Deliot, C. M. Delitzsch, M. Deliyergiyev, A. Dell’Acqua, L. Dell’Asta, M. Dell’Orso, M. Della Pietra, D. della Volpe, M. Delmastro, P. A. Delsart, C. Deluca, D. A. DeMarco, S. Demers, M. Demichev, A. Demilly, S. P. Denisov, D. Derendarz, J. E. Derkaoui, F. Derue, P. Dervan, K. Desch, C. Deterre, P. O. Deviveiros, A. Dewhurst, S. Dhaliwal, A. Di Ciaccio, L. Di Ciaccio, A. Di Domenico, C. Di Donato, A. Di Girolamo, B. Di Girolamo, A. Di Mattia, B. Di Micco, R. Di Nardo, A. Di Simone, R. Di Sipio, D. Di Valentino, C. Diaconu, M. Diamond, F. A. Dias, M. A. Diaz, E. B. Diehl, J. Dietrich, S. Diglio, A. Dimitrievska, J. Dingfelder, P. Dita, S. Dita, F. Dittus, F. Djama, T. Djobava, J. I. Djuvsland, M. A. B. do Vale, D. Dobos, M. Dobre, C. Doglioni, T. Dohmae, J. Dolejsi, Z. Dolezal, B. A. Dolgoshein, M. Donadelli, S. Donati, P. Dondero, J. Donini, J. Dopke, A. Doria, M. T. Dova, A. T. Doyle, E. Drechsler, M. Dris, E. Dubreuil, E. Duchovni, G. Duckeck, O. A. Ducu, D. Duda, A. Dudarev, L. Duflot, L. Duguid, M. Dührssen, M. Dunford, H. Duran Yildiz, M. Düren, A. Durglishvili, D. Duschinger, M. Dyndal, C. Eckardt, K. M. Ecker, R. C. Edgar, W. Edson, N. C. Edwards, W. Ehrenfeld, T. Eifert, G. Eigen, K. Einsweiler, T. Ekelof, M. El Kacimi, M. Ellert, S. Elles, F. Ellinghaus, A. A. Elliot, N. Ellis, J. Elmsheuser, M. Elsing, D. Emeliyanov, Y. Enari, O. C. Endner, M. Endo, J. Erdmann, A. Ereditato, G. Ernis, J. Ernst, M. Ernst, S. Errede, E. Ertel, M. Escalier, H. Esch, C. Escobar, B. Esposito, A. I. Etienvre, E. Etzion, H. Evans, A. Ezhilov, L. Fabbri, G. Facini, R. M. Fakhrutdinov, S. Falciano, R. J. Falla, J. Faltova, Y. Fang, M. Fanti, A. Farbin, A. Farilla, T. Farooque, S. Farrell, S. M. Farrington, P. Farthouat, F. Fassi, P. Fassnacht, D. Fassouliotis, M. Faucci Giannelli, A. Favareto, L. Fayard, P. Federic, O. L. Fedin, W. Fedorko, S. Feigl, L. Feligioni, C. Feng, E. J. Feng, H. Feng, A. B. Fenyuk, P. Fernandez Martinez, S. Fernandez Perez, J. Ferrando, A. Ferrari, P. Ferrari, R. Ferrari, D. E. Ferreira de Lima, A. Ferrer, D. Ferrere, C. Ferretti, A. Ferretto Parodi, M. Fiascaris, F. Fiedler, A. Filipčič, M. Filipuzzi, F. Filthaut, M. Fincke-Keeler, K. D. Finelli, M. C. N. Fiolhais, L. Fiorini, A. Firan, A. Fischer, C. Fischer, J. Fischer, W. C. Fisher, E. A. Fitzgerald, M. Flechl, I. Fleck, P. Fleischmann, S. Fleischmann, G. T. Fletcher, G. Fletcher, T. Flick, A. Floderus, L. R. Flores Castillo, M. J. Flowerdew, A. Formica, A. Forti, D. Fournier, H. Fox, S. Fracchia, P. Francavilla, M. Franchini, D. Francis, L. Franconi, M. Franklin, M. Fraternali, D. Freeborn, S. T. French, F. Friedrich, D. Froidevaux, J. A. Frost, C. Fukunaga, E. Fullana Torregrosa, B. G. Fulsom, J. Fuster, C. Gabaldon, O. Gabizon, A. Gabrielli, A. Gabrielli, S. Gadatsch, S. Gadomski, G. Gagliardi, P. Gagnon, C. Galea, B. Galhardo, E. J. Gallas, B. J. Gallop, P. Gallus, G. Galster, K. K. Gan, J. Gao, Y. Gao, Y. S. Gao, F. M. Garay Walls, F. Garberson, C. García, J. E. García Navarro, M. Garcia-Sciveres, R. W. Gardner, N. Garelli, V. Garonne, C. Gatti, A. Gaudiello, G. Gaudio, B. Gaur, L. Gauthier, P. Gauzzi, I. L. Gavrilenko, C. Gay, G. Gaycken, E. N. Gazis, P. Ge, Z. Gecse, C. N. P. Gee, D. A. A. Geerts, Ch. Geich-Gimbel, M. P. Geisler, C. Gemme, M. H. Genest, S. Gentile, M. George, S. George, D. Gerbaudo, A. Gershon, H. Ghazlane, B. Giacobbe, S. Giagu, V. Giangiobbe, P. Giannetti, B. Gibbard, S. M. Gibson, M. Gilchriese, T. P. S. Gillam, D. Gillberg, G. Gilles, D. M. Gingrich, N. Giokaris, M. P. Giordani, F. M. Giorgi, F. M. Giorgi, P. F. Giraud, P. Giromini, D. Giugni, C. Giuliani, M. Giulini, B. K. Gjelsten, S. Gkaitatzis, I. Gkialas, E. L. Gkougkousis, L. K. Gladilin, C. Glasman, J. Glatzer, P. C. F. Glaysher, A. Glazov, G. L. Glonti, M. Goblirsch-Kolb, J. R. Goddard, J. Godlewski, S. Goldfarb, T. Golling, D. Golubkov, A. Gomes, R. Gonçalo, J. Goncalves Pinto Firmino Da Costa, L. Gonella, S. González de la Hoz, G. Gonzalez Parra, S. Gonzalez-Sevilla, L. Goossens, P. A. Gorbounov, H. A. Gordon, I. Gorelov, B. Gorini, E. Gorini, A. Gorišek, E. Gornicki, A. T. Goshaw, C. Gössling, M. I. Gostkin, D. Goujdami, A. G. Goussiou, N. Govender, H. M. X. Grabas, L. Graber, I. Grabowska-Bold, P. Grafström, K-J. Grahn, J. Gramling, E. Gramstad, S. Grancagnolo, V. Grassi, V. Gratchev, H. M. Gray, E. Graziani, Z. D. Greenwood, K. Gregersen, I. M. Gregor, P. Grenier, J. Griffiths, A. A. Grillo, K. Grimm, S. Grinstein, Ph. Gris, J.-F. Grivaz, J. P. Grohs, A. Grohsjean, E. Gross, J. Grosse-Knetter, G. C. Grossi, Z. J. Grout, L. Guan, J. Guenther, F. Guescini, D. Guest, O. Gueta, E. Guido, T. Guillemin, S. Guindon, U. Gul, C. Gumpert, J. Guo, S. Gupta, P. Gutierrez, N. G. Gutierrez Ortiz, C. Gutschow, C. Guyot, C. Gwenlan, C. B. Gwilliam, A. Haas, C. Haber, H. K. Hadavand, N. Haddad, P. Haefner, S. Hageböck, Z. Hajduk, H. Hakobyan, M. Haleem, J. Haley, D. Hall, G. Halladjian, G. D. Hallewell, K. Hamacher, P. Hamal, K. Hamano, M. Hamer, A. Hamilton, S. Hamilton, G. N. Hamity, P. G. Hamnett, L. Han, K. Hanagaki, K. Hanawa, M. Hance, P. Hanke, R. Hanna, J. B. Hansen, J. D. Hansen, M. C. Hansen, P. H. Hansen, K. Hara, A. S. Hard, T. Harenberg, F. Hariri, S. Harkusha, R. D. Harrington, P. F. Harrison, F. Hartjes, M. Hasegawa, S. Hasegawa, Y. Hasegawa, A. Hasib, S. Hassani, S. Haug, R. Hauser, L. Hauswald, M. Havranek, C. M. Hawkes, R. J. Hawkings, A. D. Hawkins, T. Hayashi, D. Hayden, C. P. Hays, J. M. Hays, H. S. Hayward, S. J. Haywood, S. J. Head, T. Heck, V. Hedberg, L. Heelan, S. Heim, T. Heim, B. Heinemann, L. Heinrich, J. Hejbal, L. Helary, S. Hellman, D. Hellmich, C. Helsens, J. Henderson, R. C. W. Henderson, Y. Heng, C. Hengler, A. Henrichs, A. M. Henriques Correia, S. Henrot-Versille, G. H. Herbert, Y. Hernández Jiménez, R. Herrberg-Schubert, G. Herten, R. Hertenberger, L. Hervas, G. G. Hesketh, N. P. Hessey, J. W. Hetherly, R. Hickling, E. Higón-Rodriguez, E. Hill, J. C. Hill, K. H. Hiller, S. J. Hillier, I. Hinchliffe, E. Hines, R. R. Hinman, M. Hirose, D. Hirschbuehl, J. Hobbs, N. Hod, M. C. Hodgkinson, P. Hodgson, A. Hoecker, M. R. Hoeferkamp, F. Hoenig, M. Hohlfeld, D. Hohn, T. R. Holmes, M. Homann, T. M. Hong, L. Hooft van Huysduynen, W. H. Hopkins, Y. Horii, A. J. Horton, J-Y. Hostachy, S. Hou, A. Hoummada, J. Howard, J. Howarth, M. Hrabovsky, I. Hristova, J. Hrivnac, T. Hryn’ova, A. Hrynevich, C. Hsu, P. J. Hsu, S.-C. Hsu, D. Hu, Q. Hu, X. Hu, Y. Huang, Z. Hubacek, F. Hubaut, F. Huegging, T. B. Huffman, E. W. Hughes, G. Hughes, M. Huhtinen, T. A. Hülsing, N. Huseynov, J. Huston, J. Huth, G. Iacobucci, G. Iakovidis, I. Ibragimov, L. Iconomidou-Fayard, E. Ideal, Z. Idrissi, P. Iengo, O. Igonkina, T. Iizawa, Y. Ikegami, K. Ikematsu, M. Ikeno, Y. Ilchenko, D. Iliadis, N. Ilic, Y. Inamaru, T. Ince, P. Ioannou, M. Iodice, K. Iordanidou, V. Ippolito, A. Irles Quiles, C. Isaksson, M. Ishino, M. Ishitsuka, R. Ishmukhametov, C. Issever, S. Istin, J. M. Iturbe Ponce, R. Iuppa, J. Ivarsson, W. Iwanski, H. Iwasaki, J. M. Izen, V. Izzo, S. Jabbar, B. Jackson, M. Jackson, P. Jackson, M. R. Jaekel, V. Jain, K. Jakobs, S. Jakobsen, T. Jakoubek, J. Jakubek, D. O. Jamin, D. K. Jana, E. Jansen, R. W. Jansky, J. Janssen, M. Janus, G. Jarlskog, N. Javadov, T. Javůrek, L. Jeanty, J. Jejelava, G.-Y. Jeng, D. Jennens, P. Jenni, J. Jentzsch, C. Jeske, S. Jézéquel, H. Ji, J. Jia, Y. Jiang, S. Jiggins, J. Jimenez Pena, S. Jin, A. Jinaru, O. Jinnouchi, M. D. Joergensen, P. Johansson, K. A. Johns, K. Jon-And, G. Jones, R. W. L. Jones, T. J. Jones, J. Jongmanns, P. M. Jorge, K. D. Joshi, J. Jovicevic, X. Ju, C. A. Jung, P. Jussel, A. Juste Rozas, M. Kaci, A. Kaczmarska, M. Kado, H. Kagan, M. Kagan, S. J. Kahn, E. Kajomovitz, C. W. Kalderon, S. Kama, A. Kamenshchikov, N. Kanaya, M. Kaneda, S. Kaneti, V. A. Kantserov, J. Kanzaki, B. Kaplan, A. Kapliy, D. Kar, K. Karakostas, A. Karamaoun, N. Karastathis, M. J. Kareem, M. Karnevskiy, S. N. Karpov, Z. M. Karpova, K. Karthik, V. Kartvelishvili, A. N. Karyukhin, L. Kashif, R. D. Kass, A. Kastanas, Y. Kataoka, A. Katre, J. Katzy, K. Kawagoe, T. Kawamoto, G. Kawamura, S. Kazama, V. F. Kazanin, M. Y. Kazarinov, R. Keeler, R. Kehoe, J. S. Keller, J. J. Kempster, H. Keoshkerian, O. Kepka, B. P. Kerševan, S. Kersten, R. A. Keyes, F. Khalil-zada, H. Khandanyan, A. Khanov, A. G. Kharlamov, T. J. Khoo, V. Khovanskiy, E. Khramov, J. Khubua, H. Y. Kim, H. Kim, S. H. Kim, Y. Kim, N. Kimura, O. M. Kind, B. T. King, M. King, R. S. B. King, S. B. King, J. Kirk, A. E. Kiryunin, T. Kishimoto, D. Kisielewska, F. Kiss, K. Kiuchi, O. Kivernyk, E. Kladiva, M. H. Klein, M. Klein, U. Klein, K. Kleinknecht, P. Klimek, A. Klimentov, R. Klingenberg, J. A. Klinger, T. Klioutchnikova, P. F. Klok, E.-E. Kluge, P. Kluit, S. Kluth, E. Kneringer, E. B. F. G. Knoops, A. Knue, A. Kobayashi, D. Kobayashi, T. Kobayashi, M. Kobel, M. Kocian, P. Kodys, T. Koffas, E. Koffeman, L. A. Kogan, S. Kohlmann, Z. Kohout, T. Kohriki, T. Koi, H. Kolanoski, I. Koletsou, A. A. Komar, Y. Komori, T. Kondo, N. Kondrashova, K. Köneke, A. C. König, S. König, T. Kono, R. Konoplich, N. Konstantinidis, R. Kopeliansky, S. Koperny, L. Köpke, A. K. Kopp, K. Korcyl, K. Kordas, A. Korn, A. A. Korol, I. Korolkov, E. V. Korolkova, O. Kortner, S. Kortner, T. Kosek, V. V. Kostyukhin, V. M. Kotov, A. Kotwal, A. Kourkoumeli-Charalampidi, C. Kourkoumelis, V. Kouskoura, A. Koutsman, R. Kowalewski, T. Z. Kowalski, W. Kozanecki, A. S. Kozhin, V. A. Kramarenko, G. Kramberger, D. Krasnopevtsev, M. W. Krasny, A. Krasznahorkay, J. K. Kraus, A. Kravchenko, S. Kreiss, M. Kretz, J. Kretzschmar, K. Kreutzfeldt, P. Krieger, K. Krizka, K. Kroeninger, H. Kroha, J. Kroll, J. Kroseberg, J. Krstic, U. Kruchonak, H. Krüger, N. Krumnack, Z. V. Krumshteyn, A. Kruse, M. C. Kruse, M. Kruskal, T. Kubota, H. Kucuk, S. Kuday, S. Kuehn, A. Kugel, F. Kuger, A. Kuhl, T. Kuhl, V. Kukhtin, Y. Kulchitsky, S. Kuleshov, M. Kuna, T. Kunigo, A. Kupco, H. Kurashige, Y. A. Kurochkin, R. Kurumida, V. Kus, E. S. Kuwertz, M. Kuze, J. Kvita, T. Kwan, D. Kyriazopoulos, A. La Rosa, J. L. La Rosa Navarro, L. La Rotonda, C. Lacasta, F. Lacava, J. Lacey, H. Lacker, D. Lacour, V. R. Lacuesta, E. Ladygin, R. Lafaye, B. Laforge, T. Lagouri, S. Lai, L. Lambourne, S. Lammers, C. L. Lampen, W. Lampl, E. Lançon, U. Landgraf, M. P. J. Landon, V. S. Lang, J. C. Lange, A. J. Lankford, F. Lanni, K. Lantzsch, S. Laplace, C. Lapoire, J. F. Laporte, T. Lari, F. Lasagni Manghi, M. Lassnig, P. Laurelli, W. Lavrijsen, A. T. Law, P. Laycock, O. Le Dortz, E. Le Guirriec, E. Le Menedeu, M. LeBlanc, T. LeCompte, F. Ledroit-Guillon, C. A. Lee, S. C. Lee, L. Lee, G. Lefebvre, M. Lefebvre, F. Legger, C. Leggett, A. Lehan, G. Lehmann Miotto, X. Lei, W. A. Leight, A. Leisos, A. G. Leister, M. A. L. Leite, R. Leitner, D. Lellouch, B. Lemmer, K. J. C. Leney, T. Lenz, B. Lenzi, R. Leone, S. Leone, C. Leonidopoulos, S. Leontsinis, C. Leroy, C. G. Lester, M. Levchenko, J. Levêque, D. Levin, L. J. Levinson, M. Levy, A. Lewis, A. M. Leyko, M. Leyton, B. Li, H. Li, H. L. Li, L. Li, L. Li, S. Li, Y. Li, Z. Liang, H. Liao, B. Liberti, A. Liblong, P. Lichard, K. Lie, J. Liebal, W. Liebig, C. Limbach, A. Limosani, S. C. Lin, T. H. Lin, F. Linde, B. E. Lindquist, J. T. Linnemann, E. Lipeles, A. Lipniacka, M. Lisovyi, T. M. Liss, D. Lissauer, A. Lister, A. M. Litke, B. Liu, D. Liu, J. Liu, J. B. Liu, K. Liu, L. Liu, M. Liu, M. Liu, Y. Liu, M. Livan, A. Lleres, J. Llorente Merino, S. L. Lloyd, F. Lo Sterzo, E. Lobodzinska, P. Loch, W. S. Lockman, F. K. Loebinger, A. E. Loevschall-Jensen, A. Loginov, T. Lohse, K. Lohwasser, M. Lokajicek, B. A. Long, J. D. Long, R. E. Long, K. A. Looper, L. Lopes, D. Lopez Mateos, B. Lopez Paredes, I. Lopez Paz, J. Lorenz, N. Lorenzo Martinez, M. Losada, P. Loscutoff, P. J. Lösel, X. Lou, A. Lounis, J. Love, P. A. Love, N. Lu, H. J. Lubatti, C. Luci, A. Lucotte, F. Luehring, W. Lukas, L. Luminari, O. Lundberg, B. Lund-Jensen, D. Lynn, R. Lysak, E. Lytken, H. Ma, L. L. Ma, G. Maccarrone, A. Macchiolo, C. M. Macdonald, J. Machado Miguens, D. Macina, D. Madaffari, R. Madar, H. J. Maddocks, W. F. Mader, A. Madsen, S. Maeland, T. Maeno, A. Maevskiy, E. Magradze, K. Mahboubi, J. Mahlstedt, C. Maiani, C. Maidantchik, A. A. Maier, T. Maier, A. Maio, S. Majewski, Y. Makida, N. Makovec, B. Malaescu, Pa. Malecki, V. P. Maleev, F. Malek, U. Mallik, D. Malon, C. Malone, S. Maltezos, V. M. Malyshev, S. Malyukov, J. Mamuzic, G. Mancini, B. Mandelli, L. Mandelli, I. Mandić, R. Mandrysch, J. Maneira, A. Manfredini, L. Manhaes de Andrade Filho, J. Manjarres Ramos, A. Mann, P. M. Manning, A. Manousakis-Katsikakis, B. Mansoulie, R. Mantifel, M. Mantoani, L. Mapelli, L. March, G. Marchiori, M. Marcisovsky, C. P. Marino, M. Marjanovic, F. Marroquim, S. P. Marsden, Z. Marshall, L. F. Marti, S. Marti-Garcia, B. Martin, T. A. Martin, V. J. Martin, B. Martin dit Latour, M. Martinez, S. Martin-Haugh, V. S. Martoiu, A. C. Martyniuk, M. Marx, F. Marzano, A. Marzin, L. Masetti, T. Mashimo, R. Mashinistov, J. Masik, A. L. Maslennikov, I. Massa, L. Massa, N. Massol, P. Mastrandrea, A. Mastroberardino, T. Masubuchi, P. Mättig, J. Mattmann, J. Maurer, S. J. Maxfield, D. A. Maximov, R. Mazini, S. M. Mazza, L. Mazzaferro, G. Mc Goldrick, S. P. Mc Kee, A. McCarn, R. L. McCarthy, T. G. McCarthy, N. A. McCubbin, K. W. McFarlane, J. A. Mcfayden, G. Mchedlidze, S. J. McMahon, R. A. McPherson, M. Medinnis, S. Meehan, S. Mehlhase, A. Mehta, K. Meier, C. Meineck, B. Meirose, B. R. Mellado Garcia, F. Meloni, A. Mengarelli, S. Menke, E. Meoni, K. M. Mercurio, S. Mergelmeyer, P. Mermod, L. Merola, C. Meroni, F. S. Merritt, A. Messina, J. Metcalfe, A. S. Mete, C. Meyer, C. Meyer, J-P. Meyer, J. Meyer, R. P. Middleton, S. Miglioranzi, L. Mijović, G. Mikenberg, M. Mikestikova, M. Mikuž, M. Milesi, A. Milic, D. W. Miller, C. Mills, A. Milov, D. A. Milstead, A. A. Minaenko, Y. Minami, I. A. Minashvili, A. I. Mincer, B. Mindur, M. Mineev, Y. Ming, L. M. Mir, T. Mitani, J. Mitrevski, V. A. Mitsou, A. Miucci, P. S. Miyagawa, J. U. Mjörnmark, T. Moa, K. Mochizuki, S. Mohapatra, W. Mohr, S. Molander, R. Moles-Valls, K. Mönig, C. Monini, J. Monk, E. Monnier, J. Montejo Berlingen, F. Monticelli, S. Monzani, R. W. Moore, N. Morange, D. Moreno, M. Moreno Llácer, P. Morettini, M. Morgenstern, M. Morii, M. Morinaga, V. Morisbak, S. Moritz, A. K. Morley, G. Mornacchi, J. D. Morris, S. S. Mortensen, A. Morton, L. Morvaj, M. Mosidze, J. Moss, K. Motohashi, R. Mount, E. Mountricha, S. V. Mouraviev, E. J. W. Moyse, S. Muanza, R. D. Mudd, F. Mueller, J. Mueller, K. Mueller, R. S. P. Mueller, T. Mueller, D. Muenstermann, P. Mullen, Y. Munwes, J. A. Murillo Quijada, W. J. Murray, H. Musheghyan, E. Musto, A. G. Myagkov, M. Myska, O. Nackenhorst, J. Nadal, K. Nagai, R. Nagai, Y. Nagai, K. Nagano, A. Nagarkar, Y. Nagasaka, K. Nagata, M. Nagel, E. Nagy, A. M. Nairz, Y. Nakahama, K. Nakamura, T. Nakamura, I. Nakano, H. Namasivayam, R. F. Naranjo Garcia, R. Narayan, T. Naumann, G. Navarro, R. Nayyar, H. A. Neal, P. Yu. Nechaeva, T. J. Neep, P. D. Nef, A. Negri, M. Negrini, S. Nektarijevic, C. Nellist, A. Nelson, S. Nemecek, P. Nemethy, A. A. Nepomuceno, M. Nessi, M. S. Neubauer, M. Neumann, R. M. Neves, P. Nevski, P. R. Newman, D. H. Nguyen, R. B. Nickerson, R. Nicolaidou, B. Nicquevert, J. Nielsen, N. Nikiforou, A. Nikiforov, V. Nikolaenko, I. Nikolic-Audit, K. Nikolopoulos, J. K. Nilsen, P. Nilsson, Y. Ninomiya, A. Nisati, R. Nisius, T. Nobe, M. Nomachi, I. Nomidis, T. Nooney, S. Norberg, M. Nordberg, O. Novgorodova, S. Nowak, M. Nozaki, L. Nozka, K. Ntekas, G. Nunes Hanninger, T. Nunnemann, E. Nurse, F. Nuti, B. J. O’Brien, F. O’grady, D. C. O’Neil, V. O’Shea, F. G. Oakham, H. Oberlack, T. Obermann, J. Ocariz, A. Ochi, I. Ochoa, J. P. Ochoa-Ricoux, S. Oda, S. Odaka, H. Ogren, A. Oh, S. H. Oh, C. C. Ohm, H. Ohman, H. Oide, W. Okamura, H. Okawa, Y. Okumura, T. Okuyama, A. Olariu, S. A. Olivares Pino, D. Oliveira Damazio, E. Oliver Garcia, A. Olszewski, J. Olszowska, A. Onofre, P. U. E. Onyisi, C. J. Oram, M. J. Oreglia, Y. Oren, D. Orestano, N. Orlando, C. Oropeza Barrera, R. S. Orr, B. Osculati, R. Ospanov, G. Otero y Garzon, H. Otono, M. Ouchrif, E. A. Ouellette, F. Ould-Saada, A. Ouraou, K. P. Oussoren, Q. Ouyang, A. Ovcharova, M. Owen, R. E. Owen, V. E. Ozcan, N. Ozturk, K. Pachal, A. Pacheco Pages, C. Padilla Aranda, M. Pagáčová, S. Pagan Griso, E. Paganis, C. Pahl, F. Paige, P. Pais, K. Pajchel, G. Palacino, S. Palestini, M. Palka, D. Pallin, A. Palma, Y. B. Pan, E. Panagiotopoulou, C. E. Pandini, J. G. Panduro Vazquez, P. Pani, S. Panitkin, D. Pantea, L. Paolozzi, Th. D. Papadopoulou, K. Papageorgiou, A. Paramonov, D. Paredes Hernandez, M. A. Parker, K. A. Parker, F. Parodi, J. A. Parsons, U. Parzefall, E. Pasqualucci, S. Passaggio, F. Pastore, Fr. Pastore, G. Pásztor, S. Pataraia, N. D. Patel, J. R. Pater, T. Pauly, J. Pearce, B. Pearson, L. E. Pedersen, M. Pedersen, S. Pedraza Lopez, R. Pedro, S. V. Peleganchuk, D. Pelikan, H. Peng, B. Penning, J. Penwell, D. V. Perepelitsa, E. Perez Codina, M. T. Pérez García-Estañ, L. Perini, H. Pernegger, S. Perrella, R. Peschke, V. D. Peshekhonov, K. Peters, R. F. Y. Peters, B. A. Petersen, T. C. Petersen, E. Petit, A. Petridis, C. Petridou, E. Petrolo, F. Petrucci, N. E. Pettersson, R. Pezoa, P. W. Phillips, G. Piacquadio, E. Pianori, A. Picazio, E. Piccaro, M. Piccinini, M. A. Pickering, R. Piegaia, D. T. Pignotti, J. E. Pilcher, A. D. Pilkington, J. Pina, M. Pinamonti, J. L. Pinfold, A. Pingel, B. Pinto, S. Pires, M. Pitt, C. Pizio, L. Plazak, M.-A. Pleier, V. Pleskot, E. Plotnikova, P. Plucinski, D. Pluth, R. Poettgen, L. Poggioli, D. Pohl, G. Polesello, A. Policicchio, R. Polifka, A. Polini, C. S. Pollard, V. Polychronakos, K. Pommès, L. Pontecorvo, B. G. Pope, G. A. Popeneciu, D. S. Popovic, A. Poppleton, S. Pospisil, K. Potamianos, I. N. Potrap, C. J. Potter, C. T. Potter, G. Poulard, J. Poveda, V. Pozdnyakov, P. Pralavorio, A. Pranko, S. Prasad, S. Prell, D. Price, L. E. Price, M. Primavera, S. Prince, M. Proissl, K. Prokofiev, F. Prokoshin, E. Protopapadaki, S. Protopopescu, J. Proudfoot, M. Przybycien, E. Ptacek, D. Puddu, E. Pueschel, D. Puldon, M. Purohit, P. Puzo, J. Qian, G. Qin, Y. Qin, A. Quadt, D. R. Quarrie, W. B. Quayle, M. Queitsch-Maitland, D. Quilty, S. Raddum, V. Radeka, V. Radescu, S. K. Radhakrishnan, P. Radloff, P. Rados, F. Ragusa, G. Rahal, S. Rajagopalan, M. Rammensee, C. Rangel-Smith, F. Rauscher, S. Rave, T. Ravenscroft, M. Raymond, A. L. Read, N. P. Readioff, D. M. Rebuzzi, A. Redelbach, G. Redlinger, R. Reece, K. Reeves, L. Rehnisch, H. Reisin, M. Relich, C. Rembser, H. Ren, A. Renaud, M. Rescigno, S. Resconi, O. L. Rezanova, P. Reznicek, R. Rezvani, R. Richter, S. Richter, E. Richter-Was, O. Ricken, M. Ridel, P. Rieck, C. J. Riegel, J. Rieger, M. Rijssenbeek, A. Rimoldi, L. Rinaldi, B. Ristić, E. Ritsch, I. Riu, F. Rizatdinova, E. Rizvi, S. H. Robertson, A. Robichaud-Veronneau, D. Robinson, J. E. M. Robinson, A. Robson, C. Roda, S. Roe, O. Røhne, S. Rolli, A. Romaniouk, M. Romano, S. M. Romano Saez, E. Romero Adam, N. Rompotis, M. Ronzani, L. Roos, E. Ros, S. Rosati, K. Rosbach, P. Rose, P. L. Rosendahl, O. Rosenthal, V. Rossetti, E. Rossi, L. P. Rossi, R. Rosten, M. Rotaru, I. Roth, J. Rothberg, D. Rousseau, C. R. Royon, A. Rozanov, Y. Rozen, X. Ruan, F. Rubbo, I. Rubinskiy, V. I. Rud, C. Rudolph, M. S. Rudolph, F. Rühr, A. Ruiz-Martinez, Z. Rurikova, N. A. Rusakovich, A. Ruschke, H. L. Russell, J. P. Rutherfoord, N. Ruthmann, Y. F. Ryabov, M. Rybar, G. Rybkin, N. C. Ryder, A. F. Saavedra, G. Sabato, S. Sacerdoti, A. Saddique, H. F-W. Sadrozinski, R. Sadykov, F. Safai Tehrani, M. Saimpert, H. Sakamoto, Y. Sakurai, G. Salamanna, A. Salamon, M. Saleem, D. Salek, P. H. Sales De Bruin, D. Salihagic, A. Salnikov, J. Salt, D. Salvatore, F. Salvatore, A. Salvucci, A. Salzburger, D. Sampsonidis, A. Sanchez, J. Sánchez, V. Sanchez Martinez, H. Sandaker, R. L. Sandbach, H. G. Sander, M. P. Sanders, M. Sandhoff, C. Sandoval, R. Sandstroem, D. P. C. Sankey, M. Sannino, A. Sansoni, C. Santoni, R. Santonico, H. Santos, I. Santoyo Castillo, K. Sapp, A. Sapronov, J. G. Saraiva, B. Sarrazin, O. Sasaki, Y. Sasaki, K. Sato, G. Sauvage, E. Sauvan, G. Savage, P. Savard, C. Sawyer, L. Sawyer, J. Saxon, C. Sbarra, A. Sbrizzi, T. Scanlon, D. A. Scannicchio, M. Scarcella, V. Scarfone, J. Schaarschmidt, P. Schacht, D. Schaefer, R. Schaefer, J. Schaeffer, S. Schaepe, S. Schaetzel, U. Schäfer, A. C. Schaffer, D. Schaile, R. D. Schamberger, V. Scharf, V. A. Schegelsky, D. Scheirich, M. Schernau, C. Schiavi, C. Schillo, M. Schioppa, S. Schlenker, E. Schmidt, K. Schmieden, C. Schmitt, S. Schmitt, S. Schmitt, B. Schneider, Y. J. Schnellbach, U. Schnoor, L. Schoeffel, A. Schoening, B. D. Schoenrock, E. Schopf, A. L. S. Schorlemmer, M. Schott, D. Schouten, J. Schovancova, S. Schramm, M. Schreyer, C. Schroeder, N. Schuh, M. J. Schultens, H.-C. Schultz-Coulon, H. Schulz, M. Schumacher, B. A. Schumm, Ph. Schune, C. Schwanenberger, A. Schwartzman, T. A. Schwarz, Ph. Schwegler, Ph. Schwemling, R. Schwienhorst, J. Schwindling, T. Schwindt, M. Schwoerer, F. G. Sciacca, E. Scifo, G. Sciolla, F. Scuri, F. Scutti, J. Searcy, G. Sedov, E. Sedykh, P. Seema, S. C. Seidel, A. Seiden, F. Seifert, J. M. Seixas, G. Sekhniaidze, K. Sekhon, S. J. Sekula, K. E. Selbach, D. M. Seliverstov, N. Semprini-Cesari, C. Serfon, L. Serin, L. Serkin, T. Serre, M. Sessa, R. Seuster, H. Severini, T. Sfiligoj, F. Sforza, A. Sfyrla, E. Shabalina, M. Shamim, L. Y. Shan, R. Shang, J. T. Shank, M. Shapiro, P. B. Shatalov, K. Shaw, S. M. Shaw, A. Shcherbakova, C. Y. Shehu, P. Sherwood, L. Shi, S. Shimizu, C. O. Shimmin, M. Shimojima, M. Shiyakova, A. Shmeleva, D. Shoaleh Saadi, M. J. Shochet, S. Shojaii, S. Shrestha, E. Shulga, M. A. Shupe, S. Shushkevich, P. Sicho, O. Sidiropoulou, D. Sidorov, A. Sidoti, F. Siegert, Dj. Sijacki, J. Silva, Y. Silver, S. B. Silverstein, V. Simak, O. Simard, Lj. Simic, S. Simion, E. Simioni, B. Simmons, D. Simon, R. Simoniello, P. Sinervo, N. B. Sinev, G. Siragusa, A. N. Sisakyan, S. Yu. Sivoklokov, J. Sjölin, T. B. Sjursen, M. B. Skinner, H. P. Skottowe, P. Skubic, M. Slater, T. Slavicek, M. Slawinska, K. Sliwa, V. Smakhtin, B. H. Smart, L. Smestad, S. Yu. Smirnov, Y. Smirnov, L. N. Smirnova, O. Smirnova, M. N. K. Smith, M. Smizanska, K. Smolek, A. A. Snesarev, G. Snidero, S. Snyder, R. Sobie, F. Socher, A. Soffer, D. A. Soh, C. A. Solans, M. Solar, J. Solc, E. Yu. Soldatov, U. Soldevila, A. A. Solodkov, A. Soloshenko, O. V. Solovyanov, V. Solovyev, P. Sommer, H. Y. Song, N. Soni, A. Sood, A. Sopczak, B. Sopko, V. Sopko, V. Sorin, D. Sosa, M. Sosebee, C. L. Sotiropoulou, R. Soualah, P. Soueid, A. M. Soukharev, D. South, S. Spagnolo, M. Spalla, F. Spanò, W. R. Spearman, F. Spettel, R. Spighi, G. Spigo, L. A. Spiller, M. Spousta, T. Spreitzer, R. D. St. Denis, S. Staerz, J. Stahlman, R. Stamen, S. Stamm, E. Stanecka, C. Stanescu, M. Stanescu-Bellu, M. M. Stanitzki, S. Stapnes, E. A. Starchenko, J. Stark, P. Staroba, P. Starovoitov, R. Staszewski, P. Stavina, P. Steinberg, B. Stelzer, H. J. Stelzer, O. Stelzer-Chilton, H. Stenzel, S. Stern, G. A. Stewart, J. A. Stillings, M. C. Stockton, M. Stoebe, G. Stoicea, P. Stolte, S. Stonjek, A. R. Stradling, A. Straessner, M. E. Stramaglia, J. Strandberg, S. Strandberg, A. Strandlie, E. Strauss, M. Strauss, P. Strizenec, R. Ströhmer, D. M. Strom, R. Stroynowski, A. Strubig, S. A. Stucci, B. Stugu, N. A. Styles, D. Su, J. Su, R. Subramaniam, A. Succurro, Y. Sugaya, C. Suhr, M. Suk, V. V. Sulin, S. Sultansoy, T. Sumida, S. Sun, X. Sun, J. E. Sundermann, K. Suruliz, G. Susinno, M. R. Sutton, S. Suzuki, Y. Suzuki, M. Svatos, S. Swedish, M. Swiatlowski, I. Sykora, T. Sykora, D. Ta, C. Taccini, K. Tackmann, J. Taenzer, A. Taffard, R. Tafirout, N. Taiblum, H. Takai, R. Takashima, H. Takeda, T. Takeshita, Y. Takubo, M. Talby, A. A. Talyshev, J. Y. C. Tam, K. G. Tan, J. Tanaka, R. Tanaka, S. Tanaka, S. Tanaka, B. B. Tannenwald, N. Tannoury, S. Tapprogge, S. Tarem, F. Tarrade, G. F. Tartarelli, P. Tas, M. Tasevsky, T. Tashiro, E. Tassi, A. Tavares Delgado, Y. Tayalati, F. E. Taylor, G. N. Taylor, W. Taylor, F. A. Teischinger, M. Teixeira Dias Castanheira, P. Teixeira-Dias, K. K. Temming, H. Ten Kate, P. K. Teng, J. J. Teoh, F. Tepel, S. Terada, K. Terashi, J. Terron, S. Terzo, M. Testa, R. J. Teuscher, J. Therhaag, T. Theveneaux-Pelzer, J. P. Thomas, J. Thomas-Wilsker, E. N. Thompson, P. D. Thompson, R. J. Thompson, A. S. Thompson, L. A. Thomsen, E. Thomson, M. Thomson, R. P. Thun, M. J. Tibbetts, R. E. Ticse Torres, V. O. Tikhomirov, Yu. A. Tikhonov, S. Timoshenko, E. Tiouchichine, P. Tipton, S. Tisserant, T. Todorov, S. Todorova-Nova, J. Tojo, S. Tokár, K. Tokushuku, K. Tollefson, E. Tolley, L. Tomlinson, M. Tomoto, L. Tompkins, K. Toms, E. Torrence, H. Torres, E. Torró Pastor, J. Toth, F. Touchard, D. R. Tovey, T. Trefzger, L. Tremblet, A. Tricoli, I. M. Trigger, S. Trincaz-Duvoid, M. F. Tripiana, W. Trischuk, B. Trocmé, C. Troncon, M. Trottier-McDonald, M. Trovatelli, P. True, L. Truong, M. Trzebinski, A. Trzupek, C. Tsarouchas, J. C-L. Tseng, P. V. Tsiareshka, D. Tsionou, G. Tsipolitis, N. Tsirintanis, S. Tsiskaridze, V. Tsiskaridze, E. G. Tskhadadze, I. I. Tsukerman, V. Tsulaia, S. Tsuno, D. Tsybychev, A. Tudorache, V. Tudorache, A. N. Tuna, S. A. Tupputi, S. Turchikhin, D. Turecek, R. Turra, A. J. Turvey, P. M. Tuts, A. Tykhonov, M. Tylmad, M. Tyndel, I. Ueda, R. Ueno, M. Ughetto, M. Ugland, M. Uhlenbrock, F. Ukegawa, G. Unal, A. Undrus, G. Unel, F. C. Ungaro, Y. Unno, C. Unverdorben, J. Urban, P. Urquijo, P. Urrejola, G. Usai, A. Usanova, L. Vacavant, V. Vacek, B. Vachon, C. Valderanis, N. Valencic, S. Valentinetti, A. Valero, L. Valery, S. Valkar, E. Valladolid Gallego, S. Vallecorsa, J. A. Valls Ferrer, W. Van Den Wollenberg, P. C. Van Der Deijl, R. van der Geer, H. van der Graaf, R. Van Der Leeuw, N. van Eldik, P. van Gemmeren, J. Van Nieuwkoop, I. van Vulpen, M. C. van Woerden, M. Vanadia, W. Vandelli, R. Vanguri, A. Vaniachine, F. Vannucci, G. Vardanyan, R. Vari, E. W. Varnes, T. Varol, D. Varouchas, A. Vartapetian, K. E. Varvell, F. Vazeille, T. Vazquez Schroeder, J. Veatch, F. Veloso, T. Velz, S. Veneziano, A. Ventura, D. Ventura, M. Venturi, N. Venturi, A. Venturini, V. Vercesi, M. Verducci, W. Verkerke, J. C. Vermeulen, A. Vest, M. C. Vetterli, O. Viazlo, I. Vichou, T. Vickey, O. E. Vickey Boeriu, G. H. A. Viehhauser, S. Viel, R. Vigne, M. Villa, M. Villaplana Perez, E. Vilucchi, M. G. Vincter, V. B. Vinogradov, I. Vivarelli, F. Vives Vaque, S. Vlachos, D. Vladoiu, M. Vlasak, M. Vogel, P. Vokac, G. Volpi, M. Volpi, H. von der Schmitt, H. von Radziewski, E. von Toerne, V. Vorobel, K. Vorobev, M. Vos, R. Voss, J. H. Vossebeld, N. Vranjes, M. Vranjes Milosavljevic, V. Vrba, M. Vreeswijk, R. Vuillermet, I. Vukotic, Z. Vykydal, P. Wagner, W. Wagner, H. Wahlberg, S. Wahrmund, J. Wakabayashi, J. Walder, R. Walker, W. Walkowiak, C. Wang, F. Wang, H. Wang, H. Wang, J. Wang, J. Wang, K. Wang, R. Wang, S. M. Wang, T. Wang, X. Wang, C. Wanotayaroj, A. Warburton, C. P. Ward, D. R. Wardrope, M. Warsinsky, A. Washbrook, C. Wasicki, P. M. Watkins, A. T. Watson, I. J. Watson, M. F. Watson, G. Watts, S. Watts, B. M. Waugh, S. Webb, M. S. Weber, S. W. Weber, J. S. Webster, A. R. Weidberg, B. Weinert, J. Weingarten, C. Weiser, H. Weits, P. S. Wells, T. Wenaus, T. Wengler, S. Wenig, N. Wermes, M. Werner, P. Werner, M. Wessels, J. Wetter, K. Whalen, A. M. Wharton, A. White, M. J. White, R. White, S. White, D. Whiteson, F. J. Wickens, W. Wiedenmann, M. Wielers, P. Wienemann, C. Wiglesworth, L. A. M. Wiik-Fuchs, A. Wildauer, H. G. Wilkens, H. H. Williams, S. Williams, C. Willis, S. Willocq, A. Wilson, J. A. Wilson, I. Wingerter-Seez, F. Winklmeier, B. T. Winter, M. Wittgen, J. Wittkowski, S. J. Wollstadt, M. W. Wolter, H. Wolters, B. K. Wosiek, J. Wotschack, M. J. Woudstra, K. W. Wozniak, M. Wu, M. Wu, S. L. Wu, X. Wu, Y. Wu, T. R. Wyatt, B. M. Wynne, S. Xella, D. Xu, L. Xu, B. Yabsley, S. Yacoob, R. Yakabe, M. Yamada, Y. Yamaguchi, A. Yamamoto, S. Yamamoto, T. Yamanaka, K. Yamauchi, Y. Yamazaki, Z. Yan, H. Yang, H. Yang, Y. Yang, L. Yao, W-M. Yao, Y. Yasu, E. Yatsenko, K. H. Yau Wong, J. Ye, S. Ye, I. Yeletskikh, A. L. Yen, E. Yildirim, K. Yorita, R. Yoshida, K. Yoshihara, C. Young, C. J. S. Young, S. Youssef, D. R. Yu, J. Yu, J. M. Yu, J. Yu, L. Yuan, A. Yurkewicz, I. Yusuff, B. Zabinski, R. Zaidan, A. M. Zaitsev, J. Zalieckas, A. Zaman, S. Zambito, L. Zanello, D. Zanzi, C. Zeitnitz, M. Zeman, A. Zemla, K. Zengel, O. Zenin, T. Ženiš, D. Zerwas, D. Zhang, F. Zhang, J. Zhang, L. Zhang, R. Zhang, X. Zhang, Z. Zhang, X. Zhao, Y. Zhao, Z. Zhao, A. Zhemchugov, J. Zhong, B. Zhou, C. Zhou, L. Zhou, L. Zhou, N. Zhou, C. G. Zhu, H. Zhu, J. Zhu, Y. Zhu, X. Zhuang, K. Zhukov, A. Zibell, D. Zieminska, N. I. Zimine, C. Zimmermann, S. Zimmermann, Z. Zinonos, M. Zinser, M. Ziolkowski, L. Živković, G. Zobernig, A. Zoccoli, M. zur Nedden, G. Zurzolo, L. Zwalinski, ATLAS collaboration

**Affiliations:** Department of Physics, University of Adelaide, Adelaide, Australia; Physics Department, SUNY Albany, Albany, NY USA; Department of Physics, University of Alberta, Edmonton, AB Canada; Department of Physics, Ankara University, Ankara, Turkey; LAPP, CNRS/IN2P3 and Université Savoie Mont Blanc, Annecy-le-Vieux, France; High Energy Physics Division, Argonne National Laboratory, Argonne, IL USA; Department of Physics, University of Arizona, Tucson, AZ USA; Department of Physics, The University of Texas at Arlington, Arlington, TX USA; Physics Department, University of Athens, Athens, Greece; Physics Department, National Technical University of Athens, Zografou, Greece; Institute of Physics, Azerbaijan Academy of Sciences, Baku, Azerbaijan; Institut de Física d’Altes Energies and Departament de Física de la Universitat Autònoma de Barcelona, Barcelona, Spain; Institute of Physics, University of Belgrade, Belgrade, Serbia; Department for Physics and Technology, University of Bergen, Bergen, Norway; Physics Division, Lawrence Berkeley National Laboratory and University of California, Berkeley, CA USA; Department of Physics, Humboldt University, Berlin, Germany; Albert Einstein Center for Fundamental Physics and Laboratory for High Energy Physics, University of Bern, Bern, Switzerland; School of Physics and Astronomy, University of Birmingham, Birmingham, UK; Department of Physics, Bogazici University, Istanbul, Turkey; INFN Sezione di Bologna, Bologna, Italy; Physikalisches Institut, University of Bonn, Bonn, Germany; Department of Physics, Boston University, Boston, MA USA; Department of Physics, Brandeis University, Waltham, MA USA; Universidade Federal do Rio De Janeiro COPPE/EE/IF, Rio de Janeiro, Brazil; Physics Department, Brookhaven National Laboratory, Upton, NY USA; National Institute of Physics and Nuclear Engineering, Bucharest, Romania; Departamento de Física, Universidad de Buenos Aires, Buenos Aires, Argentina; Cavendish Laboratory, University of Cambridge, Cambridge, UK; Department of Physics, Carleton University, Ottawa, ON Canada; CERN, Geneva, Switzerland; Enrico Fermi Institute, University of Chicago, Chicago, IL USA; Departamento de Física, Pontificia Universidad Católica de Chile, Santiago, Chile; Institute of High Energy Physics, Chinese Academy of Sciences, Beijing, China; Laboratoire de Physique Corpusculaire, Clermont Université and Université Blaise Pascal and CNRS/IN2P3, Clermont-Ferrand, France; Nevis Laboratory, Columbia University, Irvington, NY USA; Niels Bohr Institute, University of Copenhagen, Copenhagen, Denmark; INFN Gruppo Collegato di Cosenza, Laboratori Nazionali di Frascati, Frascati, Italy; AGH University of Science and Technology, Faculty of Physics and Applied Computer Science, Krakow, Poland; Institute of Nuclear Physics, Polish Academy of Sciences, Kraków, Poland; Physics Department, Southern Methodist University, Dallas, TX USA; Physics Department, University of Texas at Dallas, Richardson, TX USA; DESY, Hamburg and Zeuthen, Germany; Institut für Experimentelle Physik IV, Technische Universität Dortmund, Dortmund, Germany; Institut für Kern- und Teilchenphysik, Technische Universität Dresden, Dresden, Germany; Department of Physics, Duke University, Durham, NC USA; SUPA, School of Physics and Astronomy, University of Edinburgh, Edinburgh, UK; INFN Laboratori Nazionali di Frascati, Frascati, Italy; Fakultät für Mathematik und Physik, Albert-Ludwigs-Universität, Freiburg, Germany; Section de Physique, Université de Genève, Geneva, Switzerland; INFN Sezione di Genova, Genova, Italy; E. Andronikashvili Institute of Physics, Iv. Javakhishvili Tbilisi State University, Tbilisi, Georgia; II Physikalisches Institut, Justus-Liebig-Universität Giessen, Giessen, Germany; SUPA, School of Physics and Astronomy, University of Glasgow, Glasgow, UK; II Physikalisches Institut, Georg-August-Universität, Göttingen, Germany; Laboratoire de Physique Subatomique et de Cosmologie, Université Grenoble-Alpes, CNRS/IN2P3, Grenoble, France; Department of Physics, Hampton University, Hampton, VA USA; Laboratory for Particle Physics and Cosmology, Harvard University, Cambridge, MA USA; Kirchhoff-Institut für Physik, Ruprecht-Karls-Universität Heidelberg, Heidelberg, Germany; Faculty of Applied Information Science, Hiroshima Institute of Technology, Hiroshima, Japan; Department of Physics, The Chinese University of Hong Kong, Shatin, NT China; Department of Physics, Indiana University, Bloomington, IN USA; Institut für Astro- und Teilchenphysik, Leopold-Franzens-Universität, Innsbruck, Austria; University of Iowa, Iowa City, IA USA; Department of Physics and Astronomy, Iowa State University, Ames, IA USA; Joint Institute for Nuclear Research, JINR Dubna, Dubna, Russia; KEK, High Energy Accelerator Research Organization, Tsukuba, Japan; Graduate School of Science, Kobe University, Kobe, Japan; Faculty of Science, Kyoto University, Kyoto, Japan; Kyoto University of Education, Kyoto, Japan; Department of Physics, Kyushu University, Fukuoka, Japan; Instituto de Física La Plata, Universidad Nacional de La Plata and CONICET, La Plata, Argentina; Physics Department, Lancaster University, Lancaster, UK; INFN Sezione di Lecce, Lecce, Italy; Oliver Lodge Laboratory, University of Liverpool, Liverpool, UK; Department of Physics, Jožef Stefan Institute and University of Ljubljana, Ljubljana, Slovenia; School of Physics and Astronomy, Queen Mary University of London, London, UK; Department of Physics, Royal Holloway University of London, Surrey, UK; Department of Physics and Astronomy, University College London, London, UK; Louisiana Tech University, Ruston, LA USA; Laboratoire de Physique Nucléaire et de Hautes Energies, UPMC and Université Paris-Diderot and CNRS/IN2P3, Paris, France; Fysiska institutionen, Lunds universitet, Lund, Sweden; Departamento de Fisica Teorica C-15, Universidad Autonoma de Madrid, Madrid, Spain; Institut für Physik, Universität Mainz, Mainz, Germany; School of Physics and Astronomy, University of Manchester, Manchester, UK; CPPM, Aix-Marseille Université and CNRS/IN2P3, Marseille, France; Department of Physics, University of Massachusetts, Amherst, MA USA; Department of Physics, McGill University, Montreal, QC Canada; School of Physics, University of Melbourne, Melbourne, VIC Australia; Department of Physics, The University of Michigan, Ann Arbor, MI USA; Department of Physics and Astronomy, Michigan State University, East Lansing, MI USA; NFN Sezione di Milano, Milan, Italy; B.I. Stepanov Institute of Physics, National Academy of Sciences of Belarus, Minsk, Republic of Belarus; National Scientific and Educational Centre for Particle and High Energy Physics, Minsk, Republic of Belarus; Department of Physics, Massachusetts Institute of Technology, Cambridge, MA USA; Group of Particle Physics, University of Montreal, Montreal, QC Canada; P.N. Lebedev Institute of Physics, Academy of Sciences, Moscow, Russia; Institute for Theoretical and Experimental Physics (ITEP), Moscow, Russia; National Research Nuclear University MEPhI, Moscow, Russia; D.V. Skobeltsyn Institute of Nuclear Physics, M.V. Lomonosov Moscow State University, Moscow, Russia; Fakultät für Physik, Ludwig-Maximilians-Universität München, Munich, Germany; Max-Planck-Institut für Physik (Werner-Heisenberg-Institut), Munich, Germany; Nagasaki Institute of Applied Science, Nagasaki, Japan; Graduate School of Science and Kobayashi-Maskawa Institute, Nagoya University, Nagoya, Japan; INFN Sezione di Napoli, Naples, Italy; Department of Physics and Astronomy, University of New Mexico, Albuquerque, NM USA; Institute for Mathematics, Astrophysics and Particle Physics, Radboud University Nijmegen/Nikhef, Nijmegen, The Netherlands; Nikhef National Institute for Subatomic Physics and University of Amsterdam, Amsterdam, The Netherlands; Department of Physics, Northern Illinois University, De Kalb, IL USA; Budker Institute of Nuclear Physics, SB RAS, Novosibirsk, Russia; Department of Physics, New York University, New York, NY USA; Ohio State University, Columbus, OH USA; Faculty of Science, Okayama University, Okayama, Japan; Homer L. Dodge Department of Physics and Astronomy, University of Oklahoma, Norman, OK USA; Department of Physics, Oklahoma State University, Stillwater, OK USA; Palacký University, RCPTM, Olomouc, Czech Republic; Center for High Energy Physics, University of Oregon, Eugene, OR USA; LAL, Université Paris-Sud and CNRS/IN2P3, Orsay, France; Graduate School of Science, Osaka University, Osaka, Japan; Department of Physics, University of Oslo, Oslo, Norway; Department of Physics, Oxford University, Oxford, UK; INFN Sezione di Pavia, Pavia, Italy; Department of Physics, University of Pennsylvania, Philadelphia, PA USA; National Research Centre “Kurchatov Institute” B.P.Konstantinov Petersburg Nuclear Physics Institute, St. Petersburg, Russia; INFN Sezione di Pisa, Pisa, Italy; Department of Physics and Astronomy, University of Pittsburgh, Pittsburgh, PA USA; Laboratorio de Instrumentacao e Fisica Experimental de Particulas, LIP, Lisbon, Portugal; Institute of Physics, Academy of Sciences of the Czech Republic, Prague, Czech Republic; Czech Technical University in Prague, Prague, Czech Republic; Faculty of Mathematics and Physics, Charles University in Prague, Prague, Czech Republic; State Research Center Institute for High Energy Physics, Protvino, Russia; Particle Physics Department, Rutherford Appleton Laboratory, Didcot, UK; Ritsumeikan University, Kusatsu, Shiga Japan; INFN Sezione di Roma, Rome, Italy; INFN Sezione di Roma Tor Vergata, Rome, Italy; INFN Sezione di Roma Tre, Rome, Italy; Faculté des Sciences Ain Chock, Réseau Universitaire de Physique des Hautes Energies-Université Hassan II, Casablanca, Morocco; DSM/IRFU (Institut de Recherches sur les Lois Fondamentales de l’Univers), CEA Saclay (Commissariat à l’Energie Atomique et aux Energies Alternatives), Gif-sur-Yvette, France; Santa Cruz Institute for Particle Physics, University of California Santa Cruz, Santa Cruz, CA USA; Department of Physics, University of Washington, Seattle, WA USA; Department of Physics and Astronomy, University of Sheffield, Sheffield, UK; Department of Physics, Shinshu University, Nagano, Japan; Fachbereich Physik, Universität Siegen, Siegen, Germany; Department of Physics, Simon Fraser University, Burnaby, BC Canada; SLAC National Accelerator Laboratory, Stanford, CA USA; Faculty of Mathematics, Physics and Informatics, Comenius University, Bratislava, Slovak Republic; Department of Physics, University of Cape Town, Cape Town, South Africa; Department of Physics, Stockholm University, Stockholm, Sweden; Physics Department, Royal Institute of Technology, Stockholm, Sweden; Departments of Physics and Astronomy and Chemistry, Stony Brook University, Stony Brook, NY USA; Department of Physics and Astronomy, University of Sussex, Brighton, UK; School of Physics, University of Sydney, Sydney, Australia; Institute of Physics, Academia Sinica, Taipei, Taiwan; Department of Physics, Technion: Israel Institute of Technology, Haifa, Israel; Raymond and Beverly Sackler School of Physics and Astronomy, Tel Aviv University, Tel Aviv, Israel; Department of Physics, Aristotle University of Thessaloniki, Thessaloníki, Greece; International Center for Elementary Particle Physics and Department of Physics, The University of Tokyo, Tokyo, Japan; Graduate School of Science and Technology, Tokyo Metropolitan University, Tokyo, Japan; Department of Physics, Tokyo Institute of Technology, Tokyo, Japan; Department of Physics, University of Toronto, Toronto, ON Canada; TRIUMF, Vancouver, BC Canada; Faculty of Pure and Applied Sciences, University of Tsukuba, Tsukuba, Japan; Department of Physics and Astronomy, Tufts University, Medford, MA USA; Centro de Investigaciones, Universidad Antonio Narino, Bogotá, Colombia; Department of Physics and Astronomy, University of California Irvine, Irvine, CA USA; INFN Gruppo Collegato di Udine, Sezione di Trieste, Udine, Italy; Department of Physics, University of Illinois, Urbana, IL USA; Department of Physics and Astronomy, University of Uppsala, Uppsala, Sweden; Instituto de Física Corpuscular (IFIC) and Departamento de Física Atómica, Molecular y Nuclear and Departamento de Ingeniería Electrónica and Instituto de Microelectrónica de Barcelona (IMB-CNM), University of Valencia and CSIC, Valencia, Spain; Department of Physics, University of British Columbia, Vancouver, BC Canada; Department of Physics and Astronomy, University of Victoria, Victoria, BC Canada; Department of Physics, University of Warwick, Coventry, UK; Waseda University, Tokyo, Japan; Department of Particle Physics, The Weizmann Institute of Science, Rehovot, Israel; Department of Physics, University of Wisconsin, Madison, WI USA; Fakultät für Physik und Astronomie, Julius-Maximilians-Universität, Würzburg, Germany; Fachbereich C Physik, Bergische Universität Wuppertal, Wuppertal, Germany; Department of Physics, Yale University, New Haven, CT USA; Yerevan Physics Institute, Yerevan, Armenia; Centre de Calcul de l’Institut National de Physique Nucléaire et de Physique des Particules (IN2P3), Villeurbanne, France; Istanbul Aydin University, Istanbul, Turkey; Division of Physics, TOBB University of Economics and Technology, Istanbul, Turkey; Department of Physics, Dogus University, Istanbul, Turkey; Department of Physics Engineering, Gaziantep University, Gaziantep, Turkey; Dipartimento di Fisica e Astronomia, Università di Bologna, Bologna, Italy; Electrical Circuits Department, Federal University of Juiz de Fora (UFJF), Juiz de Fora, Brazil; Federal University of Sao Joao del Rei (UFSJ), Sao Joao del Rei, Brazil; Instituto de Fisica, Universidade de Sao Paulo, São Paulo, Brazil; Physics Department, National Institute for Research and Development of Isotopic and Molecular Technologies, Cluj Napoca, Romania; University Politehnica Bucharest, Bucharest, Romania; West University in Timisoara, Timisoara, Romania; Departamento de Física, Universidad Técnica Federico Santa María, Valparaiso, Chile; Department of Modern Physics, University of Science and Technology of China, Anhui, China; Department of Physics, Nanjing University, Jiangsu, China; School of Physics, Shandong University, Shandong, China; Department of Physics and Astronomy, Shanghai Key Laboratory for Particle Physics and Cosmology, Shanghai Jiao Tong University, Shanghai, China; Physics Department, Tsinghua University, Beijing, 100084 China; Dipartimento di Fisica, Università della Calabria, Rende, Italy; Marian Smoluchowski Institute of Physics, Jagiellonian University, Kraków, Poland; Dipartimento di Fisica, Università di Genova, Genova, Italy; High Energy Physics Institute, Tbilisi State University, Tbilisi, Georgia; Physikalisches Institut, Ruprecht-Karls-Universität Heidelberg, Heidelberg, Germany; ZITI Institut für technische Informatik, Ruprecht-Karls-Universität Heidelberg, Mannheim, Germany; Department of Physics, The University of Hong Kong, Pok Fu Lam, China; Department of Physics, The Hong Kong University of Science and Technology, Clear Water Bay, Kowloon, Hong Kong, China; Dipartimento di Matematica e Fisica, Università del Salento, Lecce, Italy; Dipartimento di Fisica, Università di Milano, Milan, Italy; Dipartimento di Fisica, Università di Napoli, Naples, Italy; Dipartimento di Fisica, Università di Pavia, Pavia, Italy; Dipartimento di Fisica E. Fermi, Università di Pisa, Pisa, Italy; Faculdade de Ciências, Universidade de Lisboa, Lisbon, Portugal; Department of Physics, University of Coimbra, Coimbra, Portugal; Centro de Física Nuclear da Universidade de Lisboa, Lisbon, Portugal; Departamento de Fisica, Universidade do Minho, Braga, Portugal; Departamento de Fisica Teorica y del Cosmos and CAFPE, Universidad de Granada, Granada, Spain; Dep Fisica and CEFITEC of Faculdade de Ciencias e Tecnologia, Universidade Nova de Lisboa, Caparica, Portugal; Dipartimento di Fisica, Sapienza Università di Roma, Rome, Italy; Dipartimento di Fisica, Università di Roma Tor Vergata, Rome, Italy; Dipartimento di Matematica e Fisica, Università Roma Tre, Rome, Italy; Centre National de l’Energie des Sciences Techniques Nucleaires, Rabat, Morocco; Faculté des Sciences Semlalia, Université Cadi Ayyad, LPHEA-Marrakech, Marrakech, Morocco; Faculté des Sciences, Université Mohamed Premier and LPTPM, Oujda, Morocco; Faculté des Sciences, Université Mohammed V-Agdal, Rabat, Morocco; Department of Subnuclear Physics, Institute of Experimental Physics of the Slovak Academy of Sciences, Kosice, Slovak Republic; Department of Physics, University of Johannesburg, Johannesburg, South Africa; School of Physics, University of the Witwatersrand, Johannesburg, South Africa; The Oskar Klein Centre, Stockholm, Sweden; Department of Physics and Astronomy, York University, Toronto, ON Canada; ICTP, Trieste, Italy; Dipartimento di Chimica, Fisica e Ambiente, Università di Udine, Udine, Italy; CERN, Geneva, Switzerland

## Abstract

A search for heavy long-lived multi-charged particles is performed using the ATLAS detector at the LHC. Data collected in 2012 at $\sqrt{s}=8$ TeV from *pp* collisions corresponding to an integrated luminosity of 20.3 fb$^{-1}$are examined. Particles producing anomalously high ionisation, consistent with long-lived massive particles with electric charges from $|q|=2e$ to $|q|=6e$ are searched for. No signal candidate events are observed, and 95 % confidence level cross-section upper limits are interpreted as lower mass limits for a Drell–Yan production model. The mass limits range between 660 and 785 GeV.

## Introduction

This article describes a search for heavy long-lived^1^ multi-charged particles (MCPs) in $\sqrt{s}=$ 8 TeV *pp* collisions data collected in 2012 by the ATLAS detector at the CERN Large Hadron Collider (LHC). Data taken in stable beam conditions and with all ATLAS subsystems operational are used, resulting in an integrated luminosity of 20.3 fb$^{-1}$. The search is performed in the MCP mass range of 50–1000 GeV, for electric charges^2^${|q|=ze}$, with the charge numbers ${z=2}$, 3, 4, 5, and 6. The observation of such particles possessing an electric charge above the elementary charge *e* would be a signature for physics beyond the Standard Model. Several theories predict such particles, including the almost-commutative model [1], the walking technicolor model [2], and the left-right symmetric model [3], which predicts a doubly charged Higgs boson. Any observation of the particles predicted by the first two models could have implications for the formation of composite dark matter: the doubly charged particles (or, in general, particles with an even charge $|q| = 2ne$) could explain many results of experimental searches for dark matter [4]. No such particles have been observed so far in cosmic ray [5] or collider searches, including several recent searches at the Tevatron [6] and the LHC [7–9].

MCPs are highly ionising, and thus leave an abnormally large ionisation signal, $\text {d}E/\text {d}x$. A search for such particles traversing the ATLAS detector leaving a track in the inner tracking detector, and producing a signal in the muon spectrometer, is reported. A purely electromagnetic coupling, proportional to the electric charge of the MCPs, is assumed for the production model. In this model, MCPs are produced in pairs via the Drell–Yan (DY) process with only photon exchange included.

This analysis is also sensitive to fractionally charged ($z>1$, non-integer) particles, but has not been interpreted explicitly for such charges. The signal efficiency in a search for MCPs with charge numbers higher than $z=6$ is expected to be less than 5 % due to the signal particle’s low velocity. Such low efficiencies require a different approach, and corresponding model interpretations are not covered in this paper.

## The ATLAS detector

The ATLAS detector [10] covers nearly the entire solid angle around the collision point. It consists of an inner tracking detector (ID) comprising a silicon pixel detector (pixel), a silicon microstrip detector (SCT) and a transition radiation tracker (TRT). The pixel detector typically provides one precise space-point measurement per track from each of its three layers. The SCT consists of four times two layers of silicon sensors arranged with small stereo angle, typically providing eight measurements per track. The TRT, covering the pseudorapidity range $|\eta |<2.0$,^3^ is a straw-based tracking detector capable of particle identification via transition radiation and ionisation energy loss measurements [11]. A typical track crosses 32 straws. Discriminators are used to compare the signal from a straw with low and high thresholds (HT) using the TRT front-end electronics. The HT is designed to discriminate between energy depositions from transition radiation photons and the energy loss of minimum ionising particles. Roughly three times the energy deposition of a minimum ionising particle is needed for a HT hit. MCPs would produce a large number of HT hits along their trajectories due to their high level of ionisation.

The ID is surrounded by a thin superconducting solenoid providing a 2 T axial magnetic field, and by high-granularity lead–liquid argon (LAr) sampling electromagnetic calorimeters. An iron–scintillator tile calorimeter provides hadronic energy measurements in the central pseudorapidity region. The endcap and forward regions are instrumented with LAr calorimeters for electromagnetic and hadronic energy measurements. In this analysis, the calorimeters are used only as passive absorbers. The calorimeter system is surrounded by a muon spectrometer (MS) incorporating three superconducting toroidal magnet assemblies. The MS is instrumented with tracking detectors designed to measure the momenta of muons that traverse the ATLAS calorimeters. The resistive-plate chambers (RPC) in the barrel region (${|\eta | < 1.05}$) and the thin-gap chambers (TGC) in the endcaps regions (${1.05 < |\eta | < 2.4}$) provide signals for the trigger. Monitored drift tube (MDT) chambers provide typically 20–25 hits per crossing track in the pseudorapidity range ${|\eta | < 2.7}$, from which a high precision momentum measurement is derived.

The amount of material in the ID varies from one-half to two radiation lengths. The overall amount of material traversed by the MCP, which includes the calorimeters and the MS, may be as high as 75 radiation lengths. Muons typically lose 3 GeV penetrating the calorimeter system. The energy loss for MCPs with charge ${|q|=ze}$ would be $z^{2}$ times this value, i.e. up to 110 GeV for ${z=6}$.

All momentum values quoted in this paper are measured by the MS, after the energy loss in the calorimeters. Charged-particle trajectories are reconstructed using standard algorithms. Since these assume particles have $z=1$, the momenta of MCPs are underestimated by a factor *z*, as the track curvature is proportional to ${p_{\text {T}}/ z}$.

## Simulated Monte Carlo samples

Benchmark samples of simulated events with MCPs are generated for a mass of 50 GeV and for a range of masses between 100 and 1000 GeV in steps of 100 GeV, with charges *ze*, $z=2$, 3, 4, 5, and 6. Pairs of MCPs are generated via the lowest-order DY process implemented in MadGraph5 [12]. The DY production process models the kinematic distributions and determines the cross-sections used for limit setting. Typical values for the cross-sections range from hundreds of picobarns for a mass of 50 GeV down to a hundredth of a femtobarn for a mass of 1000 GeV (Fig. 8). Events are generated using the CTEQ6L1 [13] parton distribution functions, and Pythia version 8.170 [14, 15] is used for hadronisation and underlying-event generation. Simulated samples with muons from $Z\rightarrow \mu \mu$ decays are generated using Pythia version 8.170 and the CT10 [16] parton distribution functions with the AU2 tune [17]. A Geant4 simulation [18, 19] is used to model the response of the ATLAS detector. Each simulated hard scattering event is overlaid with simulated minimum bias events (“pile-up”) generated with Pythia in order to reproduce the observed distribution of the number of proton–proton collisions per bunch crossing. The simulated events are reconstructed and analysed in the same way as the experimental data.

## Candidate and event selection

Because the MCPs in this search are assumed to be long-lived and therefore traverse the entire ATLAS detector, candidates are initially selected with the MS. The search, which is restricted to the ${|\eta | < 2.0}$ pseudorapidity range, is based on an analysis of specific ionisation losses in several sub-detector systems and of the fraction of TRT straws on the track with a signal amplitude exceeding the HT. The search is logically divided into four steps: trigger and event selection, preselection, tight selection and final selection. The tight and final selection steps rely on the ionisation estimators, which are introduced in the following section. An event is considered to be a candidate event if it has at least one candidate MCP (a reconstructed particle, which satisfies all selection criteria).

### Ionisation estimators

The average specific energy loss, $\text {d}E/\text {d}x$, is described by the Bethe–Bloch formula [20]. Since a particle’s energy loss increases quadratically with its charge, an MCP would leave a very characteristic signature of high ionisation in the detector. Estimates of $\text {d}E/\text {d}x$are evaluated for the pixel, TRT and MDT sub-detector systems. All three quantities are based on an underlying measurement of time-over-threshold: the time interval where a signal amplitude exceeds a certain threshold is correlated with the deposited energy.

The significance of the $\text {d}E/\text {d}x$variable in each sub-detector is defined by comparing the observed signal, $\text{d}E/\text{d}x_{\text {track}}$, with that expected from a highly relativistic muon:

1 $$\begin{aligned} S(\text{d}E/\text{d}x) = \frac{\text{d}E/\text{d}x_{\text {track}} - \langle \text{d}E/\text{d}x_{\mu }\rangle }{\sigma (\text{d}E/\text{d}x_{\mu })}. \end{aligned}$$

Here $\langle \text{d}E/\text{d}x_{\mu }\rangle$ and $\sigma (\text{d}E/\text{d}x_{\mu })$ represent, respectively, the mean and the root-mean-square width of the $\text {d}E/\text {d}x$distribution for such muons in data. For this procedure, a control sample of muons was obtained from $Z\rightarrow \mu \mu$ events. Each muon was required to be matched to a good-quality track in the ID with $p_{\text {T}}>$ 24 GeV and ${|\eta | < 2.0}$, be isolated, i.e. to carry at least $90\,\%$ of the total $p_{\text {T}}$ within the surrounding $\Delta R<0.2$ cone, and belong to an oppositely charged pair with dimuon mass between 81 GeV and 101 GeV. These requirements effectively suppress muons from other processes reducing such backgrounds to a negligible level.

In addition to the $\text {d}E/\text {d}x$estimates, the fraction of TRT hits passing the high threshold, $f^{\text {HT}}$, is another estimator of energy loss.

**Fig. 1:**
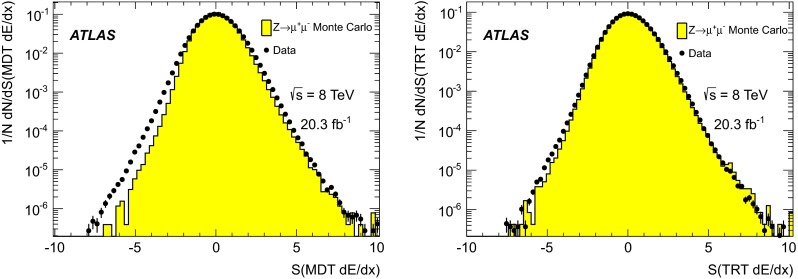
Normalised distributions of the $\text {d}E/\text {d}x$significance in the MDT, *S*(MDT $\text {d}E/\text {d}x$), (*left*) and in the TRT, *S*(TRT $\text {d}E/\text {d}x$), (*right*) for muons from $Z\rightarrow \mu \mu$ events in data and simulation

**Fig. 2:**
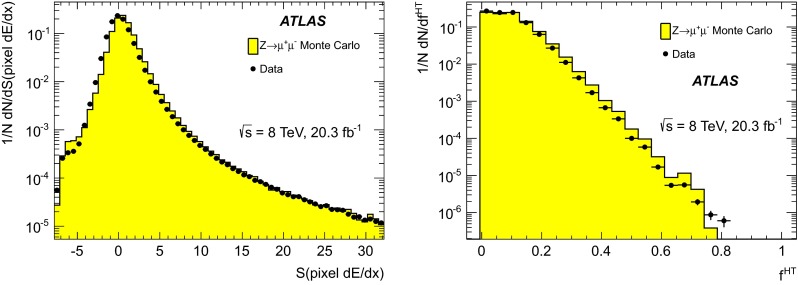
Normalised distributions of the $\text {d}E/\text {d}x$significance in the pixel system, *S*(pixel $\text {d}E/\text {d}x$), (*left*) and $f^{\text {HT}}$, the fraction of TRT hits passing the high threshold, (*right*) for muons from $Z\rightarrow \mu \mu$ events in data and simulation

In order to investigate whether the relevant variables are modelled properly, muons from $Z\rightarrow \mu \mu$ decays are compared between data and simulation. Figure 1 shows the comparison for the MDT and TRT $\text {d}E/\text {d}x$significances, and Fig. 2 for the pixel $\text {d}E/\text {d}x$significance and $f^{\text {HT}}$.

In general, Figs. 1 and 2 demonstrate good agreement between simulated and experimental data for the four selection variables. This is especially true on the high side of the distributions, which is most relevant for the analysis. The small differences observed, particularly for the *S*(MDT $\text {d}E/\text {d}x$) variable, have only minor effects on the analysis, and are accounted for as systematic uncertainties, described in Sect. 7. The behaviour of all four selection variables is found to be stable with respect to $\eta$, $\phi$ and $p_{\text {T}}$.

Detailed studies of energy loss vs. momentum distributions were performed for the pixel [21] and TRT [11] detectors, as well as for the relativistic rise domain of the Bethe–Bloch formula in the MDT. These results assure that the moderate ionisation levels (like for ${z=2}$ particles) are correctly described in the simulated data. The responses to the higher charge particles are well above the selection requirements (conservatively defined for the ${z=2}$ particles), and so the analysis is not sensitive to the precise mean position of the distributions, which may be shifted by any potential saturation effects.

### Trigger and event selection

Events collected with a single-muon trigger [22] with a transverse momentum threshold of ${p_{\text {T}}/z = 36 \mathrm{GeV}}$ are considered. This trigger is only sensitive to particles with velocity $\beta =v/c>0.6$ due to a timing window, in which particles should reach the MS. To compensate for inefficiencies in the single-muon trigger, an additional calorimeter-based trigger with a missing transverse momentum ($E_{\text {T}}^{\text {miss}}$) threshold of 80 GeV is employed. Particles reconstructed in the MS are not accounted for in the trigger $E_{\text {T}}^{\text {miss}}$ calculation, thus they contribute to the missing transverse momentum value directly. Large missing transverse momentum can also be due to an asymmetry between the energy depositions in calorimeters of the two MCPs. In case an event is selected by both of these triggers, it is assigned to the single-muon trigger for the following analysis. The $E_{\text {T}}^{\text {miss}}$ trigger recovers up to $10\,\%$ of events missed by the single-muon trigger.

Events are further required to contain at least one muon candidate with either ${p_{\text {T}}/z > 75 \mathrm{GeV}}$ (single-muon trigger) or with ${p_{\text {T}}/z > 60 \mathrm{GeV}}$ ($E_{\text {T}}^{\text {miss}}$ trigger).^4^

### Candidate track preselection

Each candidate track reconstructed in the MS with at least 7 MDT hits should match a high-quality track in the ID. Such an ID track is required to have at least 6 SCT hits and 10 TRT hits, and to originate less than 1.5 mm in both the longitudinal ($|z_{0}\sin \theta |$) and transverse ($|d_{0}|$) directions from the primary interaction point, determined via standard technique as described in Ref. [23]. Each candidate track must also be within the acceptance region of the TRT (${|\eta | < 2.0}$), have $p_{\text {T}}/z >$ 40 GeV for events collected with the single-muon trigger or $p_{\text {T}}/z >$ 30 GeV for those collected with the $E_{\text {T}}^{\text {miss}}$ trigger. The efficiency of the ID track reconstruction varies between $96\,\%$ and $98\,\%$ for all MCP charge values considered.

In order to reduce the background of high ionisation signals from two or more tracks firing the same TRT straws or MDT tubes, each candidate is required not to have an adjacent track with $p_{\text {T}}/z >$ 5 GeV within ${\Delta R< 0.01}$.

The preselected data sample (selected with these requirements) is completely dominated by muons, even in the presence of a possible signal.

### Tight selection

**Fig. 3:**
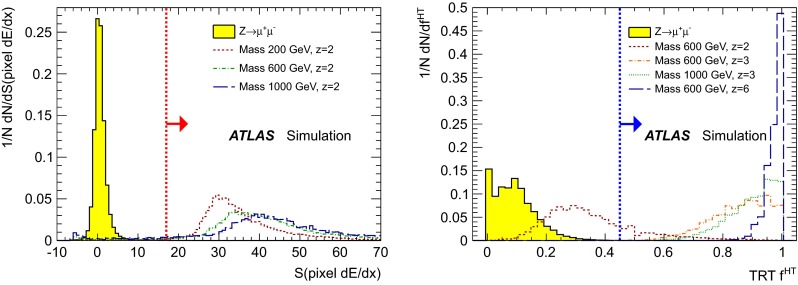
Normalised distributions of the $\text {d}E/\text {d}x$significance in the pixel system, *S*(pixel $\text {d}E/\text {d}x$), (*left*) and $f^{\text {HT}}$ (*right*) for simulated muons from $Z\rightarrow \mu \mu$ events and MCPs passing the preselection requirements. Signal distributions are shown for ${z=2}$ and masses of 200, 600 and 1000 GeV (*left*) and for ${z = 3}$ and 6 for a mass of 600 GeV and, additionally, for ${z = 3}$ and a mass of 1000 GeV (*right*). For comparison, the ${z=2}$ distribution is also shown on the *right plot*, although $f^{\text {HT}}$ is not used in the ${z=2}$ MCPs search. The *red* (*blue*) *dotted line* indicates the thresholds of the selection criteria for the ${z = 2}$ (${z \ge 3}$) case

The tight selection of highly ionising candidates is based on *S*(pixel $\text {d}E/\text {d}x$) for MCPs with ${z=2}$, and on $f^{\text {HT}}$ for MCPs with $z\ge 3$. As seen in Fig. 3, *S*(pixel $\text {d}E/\text {d}x$) is a powerful discriminator for particles with $z=2$. The signal region is defined to be the region with significance greater than 17. For higher values of *z*, the pixel readout saturates and the charge information for a particular pixel is lost. Therefore, to search for particles with ${z \ge 3}$, $f^{\text {HT}}$ (see Fig. 3) is used as a discriminating variable instead. The signal region is defined by requiring $f^{\text {HT}}$ to be above 0.45.

This tight selection using *S*(pixel $\text {d}E/\text {d}x$) or $f^{\text {HT}}$ criteria reduces the background contribution (mainly the high-$p_{\text {T}}$ muons) by almost three orders of magnitude for both the ${z = 2}$ and ${z \ge 3}$ cases, while keeping an efficiency above $95\,\%$ for the signal.

### Final selection

**Fig. 4:**
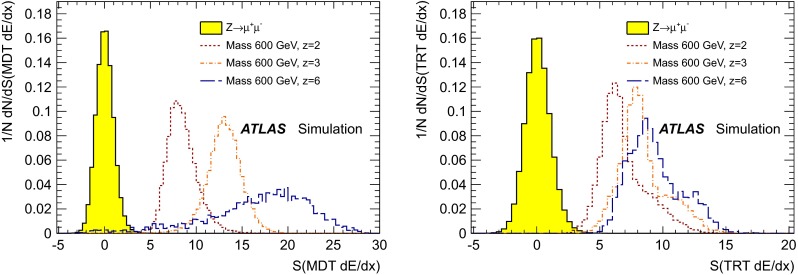
Normalised distributions of the $\text {d}E/\text {d}x$significance in the MDT, *S*(MDT $\text {d}E/\text {d}x$), (*left*) and in the TRT, *S*(TRT $\text {d}E/\text {d}x$), (*right*) for simulated muons from $Z\rightarrow \mu \mu$ events and MCPs. Signal distributions are shown for ${z=2}$, 3 and 6, for a mass of 600 GeV

In the final step of the search, *S*(MDT $\text {d}E/\text {d}x$) and *S*(TRT $\text {d}E/\text {d}x$) are used as additional discriminating variables to separate the signal and background. Figure 4 shows the distributions of these variables for simulated muons from $Z\rightarrow \mu \mu$ production compared to those of signal particles for different charges (${z=2}$, 3 and 6) and for a mass of 600 GeV. It demonstrates good separation between signal and background, which increases with increasing charge. The *S*(MDT $\text {d}E/\text {d}x$) distribution shape broadens with charge because of a larger track curvature, which hinders the track reconstruction algorithms from finding all hits on the track, thus decreasing the accuracy of the ionisation loss measurement. The detailed response for these higher charge particles may not be perfectly modelled by the simulation due to saturation effects. However, since these detectors do not lose signal at saturation, their $\text {d}E/\text {d}x$response would certainly be higher than that of ${z = 2}$ particles.

The $\text {d}E/\text {d}x$significance strongly depends on the particle’s charge and on its velocity (for a given velocity, it does not depend on the particle’s mass). For the MCPs under study, the variation of velocity ($0.6\le \beta <1$) leads to a change in $\text {d}E/\text {d}x$significances by up to $30\,\%$.

**Fig. 5:**
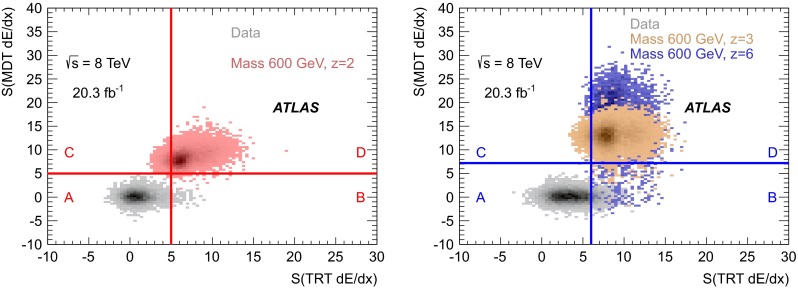
*S*(MDT $\text {d}E/\text {d}x$) versus *S*(TRT $\text {d}E/\text {d}x$) after the ${z=2}$ (*left*) or ${z \ge 3}$ (*right*) tight selection. The distributions of the data and the simulated signal samples (here for a mass of 600 GeV) are shown. The meaning of the *A, B, C* and *D* regions is discussed in the text

Two-dimensional distributions of *S*(MDT $\text {d}E/\text {d}x$) versus *S*(TRT $\text {d}E/\text {d}x$) are shown for data and simulated signal events in Fig. 5 for candidates passing the tight selection as ${z = 2}$ (left) and ${z \ge 3}$ (right), and also satisfying all previous selection criteria. As seen, the sub-detector system signatures are different for the two preselected samples, and thus the final signal regions are chosen differently. They are defined by *S*(MDT $\text {d}E/\text {d}x$) $> 5$ and *S*(TRT $\text {d}E/\text {d}x$) $> 5$ for candidates selected as ${z = 2}$ and by *S*(MDT $\text {d}E/\text {d}x$) $> 7.2$ and *S*(TRT $\text {d}E/\text {d}x$) $> 6$ for candidates selected as ${z \ge 3}$. The selection was optimised using only simulated samples and $Z\rightarrow \mu \mu$ data control samples without examining the signal region in the data.

A full summary of the analysis selections is presented in Table 1.

**Table 1:** Summary of event selection requirements for the event selections based on the single-muon trigger and the $E_{\text {T}}^{\text {miss}}$trigger

	Trigger and event selection	Candidate track selection	Tight and final selections ($z=2$)	Tight and final selections ($z\ge 3$)
Single-muon trigger case		Any muon with:		
		$$N_{\text {MDT hits}} \ge 7$$		
	$\ge 1$ trigger tight muon	$p_{\text {T}}/z > 40$ GeV		
	with $p_{\text {T}}/z >$ 36 GeV	$|\eta | < 2.0$		
		$$N_{\text {SCT hits}} \ge 6$$		
	$\ge 1$ reconstructed muon	$N_{\text {TRT hits}} \ge 10$		
	With $p_{\text {T}}/z > 75$ GeV	$|d_{0}| < 1.5\,{\mathrm{mm}}$	Event passing	Event passing
		$|z_{0}\sin \theta | < 1.5\,{\mathrm{mm}}$	preselection	preselection
		No other tracks	having a	having a
		within $\Delta R< 0.01$	muon with:	muon with:
$E_{\text {T}}^{\text {miss}}$trigger case		Any muon with:		
			*S*(pixel $\text {d}E/\text {d}x$) $> 17$	$f^{\text {HT}} > 0.45$
		$N_{\text {MDT hits}} \ge 7$	*S*(MDT $\text {d}E/\text {d}x$) $>5$	*S*(MDT $\text {d}E/\text {d}x$) $>7.2$
		$p_{\text {T}}/z >$ 30 GeV	*S*(TRT $\text {d}E/\text {d}x$) $>5$	*S*(TRT $\text {d}E/\text {d}x$) $>6$
	Trigger $E_{\text {T}}^{\text {miss}}>$ 80 GeV	$|\eta | < 2.0$		
		$$N_{\text {SCT hits}} \ge 6$$		
	$\ge 1$ reconstructed muon	$N_{\text {TRT hits}} \ge 10$		
	with $p_{\text {T}}/z >$ 60 GeV	$|d_{0}| < 1.5\,{\mathrm{mm}}$		
		$$|z_{0}\sin \theta | < 1.5\,{\mathrm{mm}}$$		
		No other tracks		
		within $\Delta R< 0.01$		

## Background estimation

The background contribution to the signal region is estimated using a method which employs sidebands of the two discriminating variables. In this method, the plane of *S*(TRT $\text {d}E/\text {d}x$) and *S*(MDT $\text {d}E/\text {d}x$) is divided into regions A, B, C and D using the final selection cuts as shown in Fig. 5. Region D is defined as the signal region, with regions A, B and C as control regions. The expected number of candidate events from background in data in region D, $N_{\text {exp}}^{\text {D}}$, is estimated from the number of observed events in data in region B after tight selection, $N_{\text {obs}}^{\text {B}}$, and the probability, *f*, to find a particle with *S*(MDT $\text {d}E/\text {d}x$) $>5$ ($7.2$) before tight selection for the ${z=2}$ (${z \ge 3}$) search case:

2 $$\begin{aligned} N_{\text {exp}}^{\text {D}} = N_{\text {obs}}^{\text {B}}\times f. \end{aligned}$$

**Fig. 6:**
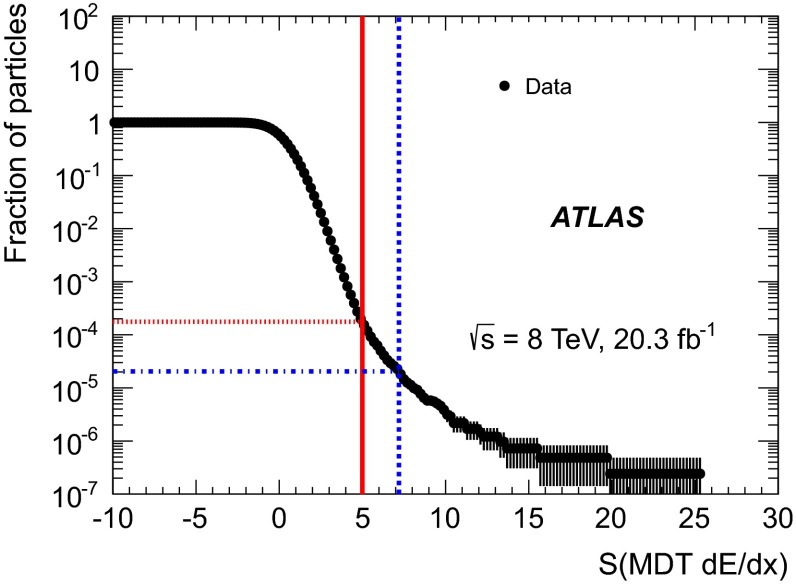
Cumulative (from above) *S*(MDT $\text {d}E/\text {d}x$) distribution before tight selection used to calculate the probability *f* to find a muon above a certain *S*(MDT $\text {d}E/\text {d}x$) value. Indicated in *red* and *blue* are the probabilities for *S*(MDT $\text {d}E/\text {d}x$) to exceed the values 5 and 7.2, respectively

The probability *f* to find a particle above some *S*(MDT $\text {d}E/\text {d}x$) value before tight selection is derived from the cumulative *S*(MDT $\text {d}E/\text {d}x$) distribution for preselected candidates in data shown in Fig. 6. Although there are no limitations on the *S*(TRT $\text {d}E/\text {d}x$) values of these particles, any possible signal contamination in this distribution is negligible.

This method relies on the fact that *S*(MDT $\text {d}E/\text {d}x$) is not correlated with the tight selection quantities, *S*(pixel $\text {d}E/\text {d}x$), $f^{\text {HT}}$ or with *S*(TRT $\text {d}E/\text {d}x$). A check was performed to demonstrate the absence of such correlations: the distributions of *S*(pixel $\text {d}E/\text {d}x$), $f^{\text {HT}}$ and *S*(TRT $\text {d}E/\text {d}x$) for muons with low *S*(MDT $\text {d}E/\text {d}x$) values were compared with those for muons with high *S*(MDT $\text {d}E/\text {d}x$) values. Excellent agreement between the two cases shows that there are no correlations between ionisation estimators in different ATLAS sub-detectors for background.

Table 2 gives numbers of observed events with particles in the B and D regions, as well as the probabilities to find a particle above certain *S*(MDT $\text {d}E/\text {d}x$) values before tight selection. The expected numbers of background events are given in the last column. They amount to $0.013$ $\pm$ $0.002$ in the signal region for the ${z = 2}$ selection and $0.026$ $\pm$ $0.003$ for the ${z \ge 3}$ selection, where the quoted uncertainties are statistical. Systematic uncertainties on the background estimate are discussed in Sect. 7.

**Table 2:** The observed event yield in data in the B region, the probability *f* to find a particle above the respective *S*(MDT $\text {d}E/\text {d}x$) value before tight selection and the expected background yield in the signal region D with its statistical uncertainty. The last column shows the observed event yield in the D region

	$N_{\text {obs}}^{\text {B}}$	*f*	$N_{\text {exp}}^{\text {D}}$	$N_{\text {obs}}^{\text {D}}$
$z=2$	76	$1.8 \times 10^{-4}$	$0.013$ $\pm$ $0.002$	0
$z \ge 3$	1251	$2.1 \times 10^{-5}$	$0.026$ $\pm$ $0.003$	0

## Signal efficiency

**Fig. 7:**
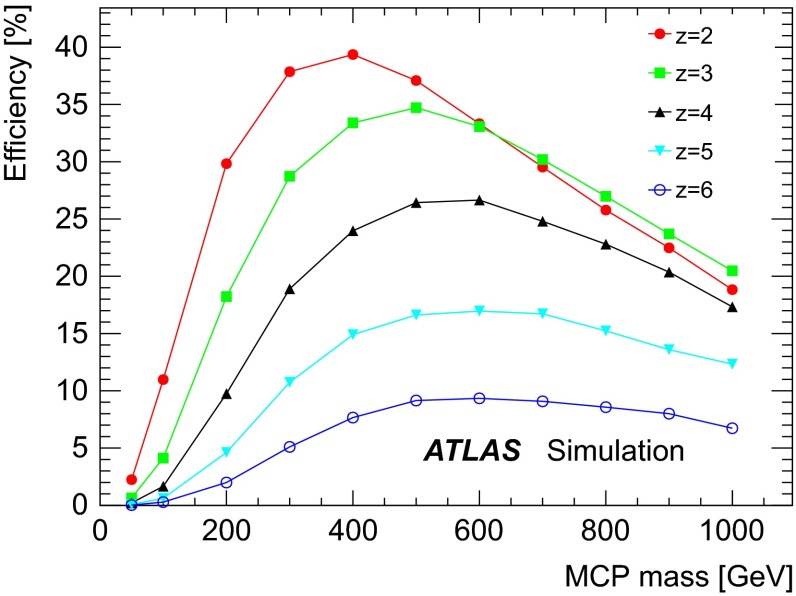
The signal efficiencies for different MCP masses and charges for the DY production model

The cross-section is given by

3 $$\begin{aligned} \sigma = \frac{N_{\text {obs}}^{\text {D}}-N_{\text {exp}}^{\text {D}}}{\mathcal {L}\times \varepsilon }, \end{aligned}$$

where $\mathcal {L}$ is the integrated luminosity of the analysed data and the numerator is the number of candidate events above the expected background. The signal efficiency, $\varepsilon$, includes trigger, reconstruction and selection efficiencies. The signal efficiency, as estimated from simulation, is shown in Fig. 7 for each signal sample.

Several factors contribute to the efficiency dependence on mass and charge. For low masses, the minimum ${p_{\text {T}}/ z}$ requirements are the main source of efficiency loss. At higher masses, the requirement to reach the MS with a $\beta$ which satisfies the trigger timing window is the primary reason for the reduction in efficiency. Also, high ionisation loss makes particles slow down: they may not fit the trigger timing window or may lose all their kinetic energy before reaching the MS. The charge dependence of the efficiency results from the higher ionisation loss and the higher effective $p_{\text {T}}$ selection, which are augmented by factors $z^2$ and *z*, respectively. For MCPs that do not reach the MS, the $E_{\text {T}}^{\text {miss}}$ would be larger for heavier MCPs and therefore more likely to fire the $E_{\text {T}}^{\text {miss}}$trigger, although the probability for such events to satisfy all selection criteria is smaller since only one candidate of an MCP pair is reconstructed in the MS.

The fraction of signal events satisfying cumulative selection requirements is given in Table 3 for several examples.

**Table 3:** Fractions of signal events (in %) with at least one MCP, which satisfy the given requirements. The uncertainties quoted are statistical

Signal benchmark point	Trigger	Preselection	Tight selection	Final selection
*m* = 100 GeV, $z=2$	$13.7\pm 0.2$	$12.8\pm 0.2$	$12.6\pm 0.2$	$11.0\pm 0.2$
*m* = 500 GeV, $z=2$	$62.8\pm 0.4$	$42.9\pm 0.3$	$39.4\pm 0.3$	$37.1\pm 0.3$
*m* = 900 GeV, $z=2$	$35.2\pm 0.4$	$26.6\pm 0.3$	$24.4\pm 0.3$	$22.5\pm 0.3$
*m* = 100 GeV, $z=4$	$2.01\pm 0.09$	$1.74\pm 0.08$	$1.71\pm 0.08$	$1.66\pm 0.08$
*m* = 500 GeV, $z=4$	$32.5\pm 0.3$	$28.7\pm 0.3$	$28.2\pm 0.3$	$26.4\pm 0.3$
*m* = 900 GeV, $z=4$	$29.7\pm 0.4$	$22.4\pm 0.3$	$21.8\pm 0.3$	$20.4\pm 0.3$
*m* = 50 GeV, $z=6$	$0.04\pm 0.02$	$0.03\pm 0.02$	$0.03\pm 0.02$	$0.02\pm 0.01$
*m* = 100 GeV, $z=6$	$0.58\pm 0.08$	$0.35\pm 0.05$	$0.32\pm 0.04$	$0.28\pm 0.04$
*m* = 500 GeV, $z=6$	$16.2\pm 0.4$	$10.3\pm 0.3$	$10.0\pm 0.2$	$9.2\pm 0.2$
*m* = 900 GeV, $z=6$	$17.4\pm 0.6$	$9.5\pm 0.4$	$9.0\pm 0.3$	$8.0\pm 0.2$

## Systematic uncertainties

Systematic uncertainties of the analysis comprise the uncertainty on the background estimate, on the signal selection efficiency, on the luminosity, and the one due to the size of the Monte Carlo samples used.

### Background estimation uncertainty

A difference is assessed between the current method and an alternate method (ABCD method, as used in Ref. [8]) where the number of expected events from background is calculated from the numbers of observed events in the three control regions according to

4 $$\begin{aligned} N_{\text {exp}}^{\text {D}} = \frac{N_{\text {obs}}^{\text {B}}\times N_{\text {obs}}^{\text {C}}}{N_{\text {obs}}^{\text {A}}}. \end{aligned}$$

Both methods use the same underlying idea, that the background estimate is proportional to the number of observed events in the region B, $N_{\text {obs}}^{\text {B}}$. However, the methods to derive the proportionality constant are different, cf. Eq. (2) and Eq. (4).

Since the ABCD method gives a large statistical uncertainty in the case of zero events in one of the control regions, the cuts on *S*(MDT $\text {d}E/\text {d}x$) were loosened from $5$ or $7.2$ down to 3 for both the ${z=2}$ and ${z \ge 3}$ selections to minimise this uncertainty, and the numbers of events expected from the background were re-estimated using the two aforementioned methods. The background estimates from the two methods were found to differ by about $25\,\%$ for both the ${z=2}$ and ${z\ge 3}$ cases, corresponding for both to a statistical significance of less than two sigma. Hence, a $25\,\%$ systematic uncertainty on the background estimate was assigned for both the ${z=2}$ and ${z\ge 3}$ cases.

### Trigger efficiency uncertainty

The uncertainty on the muon trigger efficiency has two sources: a global uncertainty on the muon trigger efficiency of 1 % [22] and a $\beta$-dependent uncertainty. The $\beta$-dependent part originates from uncertainties on the modelling of the muon trigger timing for particles with $\beta < 1$. In order to improve the description of the trigger simulation, parameterised corrections were applied. To assess the uncertainties, the parameters of these corrections were varied. The $\beta$ value of particles was varied between the true generated value and the one reconstructed in the MS from the known mass and measured momentum.^5^ The time interval needed for a signal particle to reach the RPC trigger planes was varied by the root-mean-square width of the timing distribution for muons measured in the full $Z\rightarrow \mu \mu$ sample in data. The combination of these effects ranges from $0.4\,\%$ to $13\,\%$. The timing in the TGC for data and simulation is in good agreement, and the systematic uncertainty for the TGC timing correction is negligible.

The uncertainty on the $E_{\text {T}}^{\text {miss}}$ trigger efficiency consists of two parts: a global $5\,\%$ uncertainty due to a difference between triggering in data and simulation [24] especially in the turn-on region, and $8.5\,\%$ uncertainty due to the fact that the $E_{\text {T}}^{\text {miss}}$ trigger efficiency depends on the amount of initial- and final-state radiation [25], affecting the number of signal events which pass the $E_{\text {T}}^{\text {miss}}$ trigger requirements. Varying the amount of radiation in the MC, the number of jets in an event was altered, and the relative difference of the $E_{\text {T}}^{\text {miss}}$ trigger efficiency was taken as a systematic uncertainty.

### Uncertainties due to selection

The uncertainty on the selection efficiency is evaluated by varying the requirement values used in the analysis. Several reasons motivate these variations. For example, the uncertainty on the amount of material in front of the MS, which is found to be about $1\,\%$ [26], propagates into an uncertainty on the selection efficiency due to the slowing down of particles, and its effect is covered by the effect of varying the $p_{\text {T}}$ requirement. The following variations of the nominal requirements are studied: $p_{\text {T}}$ value by $\pm 3$ % because of an uncertainty on the track $p_{\text {T}}$ measurements and the uncertainty on the amount of material; $f^{\text {HT}}$ value by $\pm 25$ % due to pile-up dependence, *S*(pixel $\text {d}E/\text {d}x$) by $\pm 10$ %, *S*(TRT $\text {d}E/\text {d}x$) by $\pm 5$ % and *S*(MDT $\text {d}E/\text {d}x$) by $\pm 15$ % because of the observed disagreement of the mean and root-mean-square width of these distributions in the $Z\rightarrow \mu \mu$ events in data and simulation, as well as of any potential mismodelling of these ionisation estimators.

For all other variables the variations have no observable effect in any of the signal samples. The total systematic uncertainties on the efficiency arising from these variations range between $1\,\%$ and $17\,\%$, where the larger uncertainty corresponds to lower-mass signal samples. This uncertainty is dominated by the effect of the $p_{\text {T}}$ requirement variation, which the lightest MCPs are most sensitive to.

The uncertainties due to the choice of parton distribution functions and due to higher orders corrections propagate into a small uncertainty on the selection efficiency, which lies well within its statistical uncertainty.

### Summary of systematic uncertainties

The contributions from the separate sources of systematic uncertainty on the signal efficiency are shown in Table 4 for several charges and mass points. The uncertainties on the luminosity and due to limited Monte Carlo samples size are also shown. Since the expected number of events from background is close to zero, the $25\,\%$ uncertainty on this number has a very small effect on the calculation of the upper limit on the cross-section. Thus, the trigger and selection efficiencies are the main sources of uncertainty. An additional statistical uncertainty to take into account the limited size of the Monte Carlo samples is added. The samples with a mass of 50 GeV and charge numbers $z=5$, $z=6$ were produced with a selection at the generator level requiring ${p_{\text {T}}/z > }$ 20 GeV in order to decrease this uncertainty. Generally, it is about $3\,\%$, although it makes a significant contribution (up to $60\,\%$) for high-charge and low-mass samples.

The uncertainty on the integrated luminosity is $2.8\,\%$. It is derived, following the same methodology as that detailed in Ref. [27], from a calibration of the luminosity scale derived from beam-separation scans performed in November 2012.

**Table 4:** Overview of separate contributions (in %) to the systematic uncertainty on the signal. The total uncertainty is given by the quadratic sum of the individual uncertainties

Signal benchmark point	Trigger efficiency	Selection efficiency	Limited Monte Carlo samples size	Luminosity	Total uncertainty
*m* = 100 GeV, $z=2$	6.1	11	1.8	2.8	13
*m* = 500 GeV, $z=2$	8.9	4.7	0.8	2.8	11
*m* = 900 GeV, $z=2$	9.7	1.8	1.2	2.8	10
*m* = 100 GeV, $z=4$	3.9	8.5	5.1	2.8	11
*m* = 500 GeV, $z=4$	9.7	2.9	1.1	2.8	11
*m* = 900 GeV, $z=4$	8.9	1.3	1.3	2.8	9.5
*m* = 50 GeV, $z=6$	4.0	13	60	2.8	61
*m* = 100 GeV, $z=6$	4.0	17	13	2.8	22
*m* = 500 GeV, $z=6$	11	4.1	2.0	2.8	12
*m* = 900 GeV, $z=6$	10	3.0	2.2	2.8	11

## Results

No signal candidate events are found for either the ${z=2}$ or the ${z \ge 3}$ selections. The results are consistent with the expectation of $0.013$ $\pm$ $0.002$(stat.) $\pm$ $0.003$(syst.) and $0.026$ $\pm$ $0.003$(stat.) $\pm$ $0.007$(syst.) background events, respectively. Since the number of signal events expected from background is very small and consistent with the observation of zero candidate events, observed and expected limits are virtually identical. The limits are computed with MCLimit [28]. It uses the CL$_s$ method [29] to discriminate between the background-only hypothesis and the signal-plus-background hypothesis, and determines exclusion limits for various MCP scenarios. The signal selection efficiency, luminosity, their uncertainties and number of observed events are taken as input for pseudo-experiments, resulting in an observed limit at $95\,\%$ confidence level (CL).

**Fig. 8:**
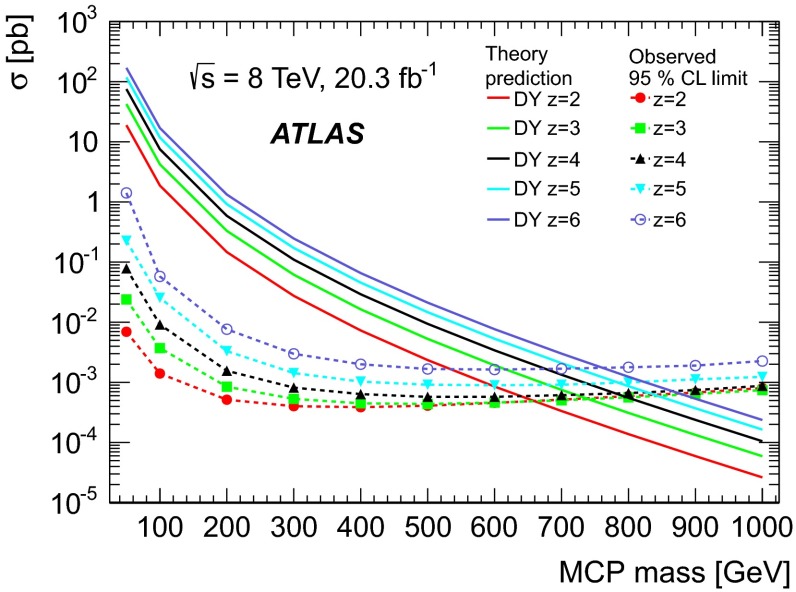
Observed $95\,\%$ CL cross-section upper limits and theoretical cross-sections as functions of the MCP’s mass for values of *z* between 2 and 6

The measurement excludes the DY model of MCP pair-production over wide ranges of tested masses. Figure 8 shows the observed $95\,\%$ CL cross-section limits as a function of mass for the five different charges. At the lowest mass values the cross-section limit ranges from 7 fb for $z=2$ to 1.4 pb for $z=6$. The most stringent cross-section limits are obtained for masses of about 400 GeV and range from 0.4 to 1.6 fb. In addition, the theoretical cross-section is shown for the simplified Drell–Yan model. The uncertainty on the theoretical cross-section is due to the parton distribution functions choice and is estimated to be $5\,\%$. For this model, the cross-section limits can be transformed into mass exclusion regions from 50 GeV up to limits of 660, 740, 780, 785, and 760 GeV for charge numbers ${z = 2}$, 3, 4, 5, and 6, respectively. Mass limits are obtained from the intersection of the observed limits and the central values of the theoretical cross-section. This result is similar to that obtained by the CMS collaboration [9] and extends the excluded region approximately 300 GeV further than in the previous ATLAS search [8].

## Conclusion

This article reports on a search for long-lived multi-charged particles produced in proton–proton collisions with the ATLAS detector at the LHC. The search uses a data sample with a center-of-mass-energy of $\sqrt{s}=$ 8 TeV and an integrated luminosity of 20.3 fb$^{-1}$. Particles with electric charges from ${|q|=2e}$ to ${|q|=6e}$ penetrating the full ATLAS detector and producing anomalously high ionisation signals in multiple detector elements are searched for. Less than one background event is expected and no events are observed. Upper limits are derived on the production cross-sections and are interpreted as mass exclusion limits for a Drell–Yan production model from 50 GeV up to 660, 740, 780, 785, and 760 GeV for charges ${|q| = 2e}$, 3*e*, 4*e*, 5*e*, and 6*e*, respectively.

## Footnotes

1 The term long-lived in this paper refers to a particle that does not decay within the ATLAS detector and penetrates its full depth.

2 Wherever a charge is quoted for the exotic particles, the charge conjugate state is also implied.

3 ATLAS uses a right-handed coordinate system with its origin at the nominal interaction point (IP) in the centre of the detector and the *z*-axis along the beam pipe. The *x*-axis points from the IP to the centre of the LHC ring, and the *y*-axis points upward. Cylindrical coordinates $(r,\phi )$ are used in the transverse plane, $\phi$ being the azimuthal angle around the *z*-axis. The pseudorapidity is defined in terms of the polar angle $\theta$ as $\eta =-\ln \tan (\theta /2)$. Angular distance is measured in units of $\Delta R \equiv \sqrt{(\Delta \eta )^{2} + (\Delta \phi )^{2}}$.

4 Information on the MDT and TRT $\text {d}E/\text {d}x$is not available in the standard ATLAS data stream. Hence, this analysis is based on special streams which include this information. The $p_{\text {T}}$ requirements for muons given here are imposed in the preparation of these streams and are not optimised for the current analysis.

5 The relation between a particle’s $\beta$, $p_{\text {T}}$ and mass, *m*, is given by $\beta = \frac{p_{\text {T}}/\sin \theta }{\sqrt{\left( p_{\text {T}}/\sin \theta \right) ^2+m^2}}$.
